# A novel prognostic and therapeutic target biomarker based on necroptosis-related gene signature and immune microenvironment infiltration in gastric cancer

**DOI:** 10.3389/fgene.2022.953997

**Published:** 2022-08-25

**Authors:** Dao Xin, Yuxin Man, Yalan Yang, Feng Wang

**Affiliations:** ^1^ Department of Oncology, The First Affiliated Hospital of Zhengzhou University, Zhengzhou, China; ^2^ Department of Medical Oncology, Sichuan Cancer Hospital, Medical School of University of Electronic Science and Technology of China, Chengdu, China

**Keywords:** necroptosis, prognostic model, cancer subtypes, gastric cancer, tumor immune microenvironment, immunotherapy

## Abstract

**Background:** Gastric cancer is a major global public health burden worldwide. Although treatment strategies are continuously improving, the overall prognosis remains poor. Necroptosis is a newly discovered form of cell death associated with anti-tumor immunity.

**Methods:** Gastric cancer (GC) data from The Cancer Genome Atlas (TCGA) and Gene Expression Omnibus (GEO) were downloaded. Bioinformatics analysis was performed to construct a necroptosis-related risk model and to establish cancer subtypes. Potential associations of the tumor immune microenvironment and immunotherapy response with necroptosis-related prognostic risk score (NRG risk score) were comprehensively explored. 16 GC and paired normal tissues were collected and RT-PCR was performed to examine expression of NRG related genes.

**Results:** GC samples were stratified into three subtypes according to prognostic necroptosis gene expression. A necroptosis risk model based on 12 genes (*NPC1L1, GAL*, *RNASE1*, *PCDH7*, *NOX4*, *GJA4*, *SLC39A4*, *BASP1*, *BLVRA*, *NCF1*, *PNOC*, and *CCR5*) was constructed and validated. The model was significantly associated with the OS and PFS of GC patients and the tumor immune microenvironment including immune cell infiltration, microsatellite instability (MSI) status, tumor mutational burden (TMB) score, immune checkpoint, and human leukocyte antigen (HLA) gene expression. A prognostic nomogram based on the NRG_score was additionally constructed. A low NRG risk score was correlated with high tumor immunogenicity and might benefit from immunotherapy.

**Conclusion:** We have identified a useful prognostic model based on necroptosis-related genes in GC and comprehensively the relationship between necroptosis and tumor immunity. Predicting value to immunotherapy response is promising, and further research to validate the model in clinical practice is needed.

## Introduction

Gastric cancer (GC), including gastroesophageal junction (GEJ) adenocarcinoma, is the fourth leading cause of cancer-related mortality worldwide (Sung et al., 2021). Adenocarcinoma of the stomach (STAD) is the most common pathological subtype, accounting for up to 95%, and other pathological types such as lymphoma and leiomyosarcoma are relatively rare. The majority of GC patients present with distant metastasis at first diagnosis. The treatment options for advanced GC have undergone significant evolution over recent years, developing from traditional chemotherapy to targeted therapy and immunotherapy, resulting in the progressive improvement of outcomes (Joshi and Badgwell, 2021). However, GC is a highly heterogeneous malignant tumor, and in clinical practice, a large number of patients do not benefit from targeted therapy or immunotherapy (Seeneevassen et al., 2021). Selection of the patient populations that potentially benefit from these treatments is therefore critical for the optimization of survival outcomes.

Necroptosis, formerly considered an unregulated accidental cell death process, is a caspase-independent form of cell death involving receptor-interacting protein kinase 1 (RIP1), RIP3, and mixed lineage kinase domain-like protein (MLKL) (Gong et al., 2019; Khoury et al., 2020). This newly discovered pathway regulates necrosis and is induced by death receptors, interferon, Toll-like receptors, intracellular RNA and DNA sensors, and other potential regulators (Pasparakis and Vandenabeele, 2015). Recent studies have provided exciting novel insights into the regulatory mechanisms of necrosis and their associations and suggest that necrosis is critical in the pathogenesis of multiple human diseases. Rather than the formation of apoptotic bodies, which occurs during cellular apoptosis, necroptosis is accompanied by rupture of the cell membrane and release of tumor neoantigens, which can trigger strong inflammatory and anti-tumor immune responses. This pathway is involved in the development and progression of various tumors and appears to serve as an effective biomarker of survival outcome or therapeutic effect (Gong et al., 2019; Sprooten et al., 2020; Tang et al., 2020). To date, limited studies have focused on the potential involvement and underlying molecular mechanisms and therapeutic response of the immunotherapy of necroptosis in gastric cancer, and few necroptosis-related cancer subtypes or prognostic models are currently available.

In this study, GC samples were collected from TCGA and GEO databases for comprehensive analysis of necroptosis-related gene (NRG) expression, mutation status, and copy number variations. Based on NRG expression patterns, all samples were classified into two necroptosis-associated subtypes. According to differentially expressed gene (DEG) patterns between the two groups, samples were classified into three different gene subtypes. We successfully constructed a predictive model and comprehensively evaluated the correlations between different risk layers and the tumor immune microenvironment (TIME). Our collective findings provide novel insights that could aid in the evolution of strategies for accurate classification and effective immunotherapy and innovative targeted therapy of gastric cancer.

## Materials and methods

### Data collection

**FIGURE 1:**
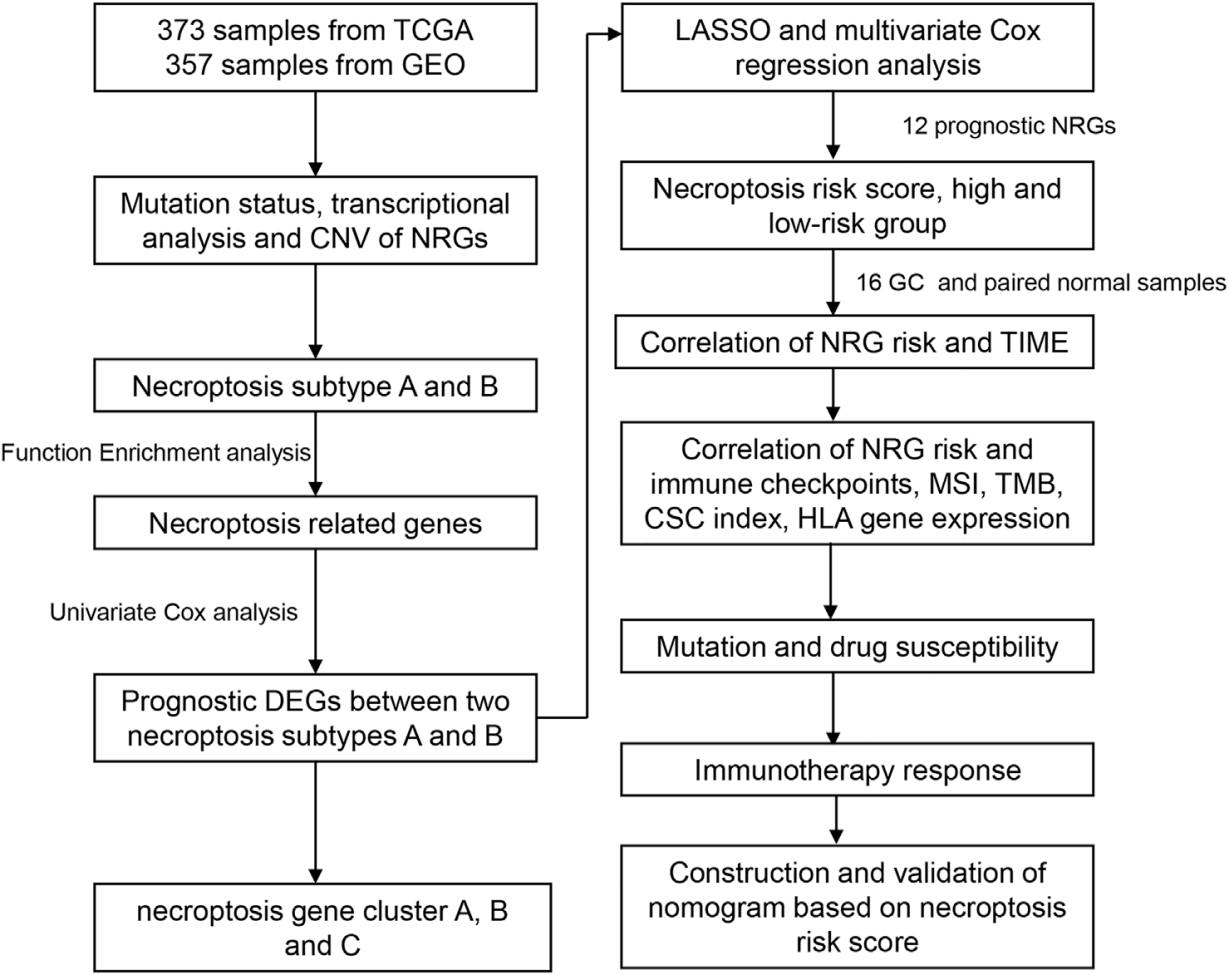
Flowchart of the study.

The flow chart of the study is presented in Figure 1. RNA sequencing data (fragments per kilobase million; FPKM) and relevant clinical and follow-up information were obtained from TCGA and GEO datasets (GSE84433). GEO database are available in GPL6947 platform (Illumina HumanHT-12 V3.0 expression beadchip). FPKM values of TCGA-stomach adenocarcinoma (STAD) data were converted and normalized to transcripts per kilobase million (TPM), which are more similar to those resulting from microarrays and more comparable between samples. Intersection genes of TCGA and GEO databases were selected out. The normal samples in TCGA database were removed and log2 was performed for GEO database. The “limma” R package was used for data normalization. The “ComBat” algorithm of “sva” R package was used to correct the batch effects due to the non-biological technical bias. Then, data from the two cohorts were combined, with the exclusion of cases with missing follow-up information or unknown survival status (Song et al., 2021; Yang et al., 2022). Clinical data, such as TNM stage, age, gender, follow-up time, and survival status, were collected. A list of 67 necroptosis-related genes (NRG) were selected for analysis (Supplementary Table S1) from the Gene Set Enrichment Analysis (GSEA) database and previous publications.

### Somatic mutations and copy number alterations of NRGs

Somatic mutation status (workflow type: VarScan2 Variant Aggregation and Masking) and copy number variation (CNV) data were downloaded from the TCGA database. Summary analysis of somatic mutation frequency in the 12 necroptosis genes was performed to select genes with high mutational frequency. Additionally, somatic copy number alterations of necroptosis genes were analyzed, along with correlations of CNV and mRNA expression and chromosomal location of necroptosis genes.

### Consensus clustering analysis of NRGs

The R package “ConsensusClusterPlus” was employed for subsequent consensus unsupervised sample clustering analysis. GC samples were classified into two different subtypes based on necroptosis genes expression. To achieve the optimal subtyping effect, cumulative distribution function (CDF) curves were increased gradually and smoothly, and sample sizes were relatively balanced among the different subgroups.

### Correlation of necroptosis subtypes with clinicopathological characteristics and prognosis

We created a heatmap to evaluate the associations of the two necroptosis subtypes with prognosis and clinicopathological characteristics, including TNM stage, pathological grade, age, and sex. Kaplan–Meier analysis was performed to compare differential prognosis in overall survival (OS). Univariate Cox regression was conducted for preliminary analysis of the correlation between the expression of individual necroptosis genes and the prognosis of GC patients.

### DEG identification and functional analysis

Differentially expressed genes (DEG) were selected between the different necroptosis subgroups using the R package “limma” at a threshold *p*-value<0.05 and a fold change of 1.5. Subsequent Gene set variation analysis (GSVA) was performed to identify the functions and biological processes of the different subgroups. GSVA was based on the hallmark gene set (c2.cp.kegg.v7.4.symbols.gmt) downloaded from the MSigDB database. Additionally, functional enrichment analysis was conducted based on DEGs using the R packages “clusterProfiler” and “enrichplot.”

### Necroptosis-related gene prognostic risk score

Necroptosis-related prognostic risk score (NRG_score) was created to quantify the necroptosis patterns of each GC sample. DEGs associated with OS were selected according to univariate Cox regression. Next, all patients were divided into different necroptosis gene clusters (A, B, C) following the unsupervised clustering method according to prognostic DEGs. All GC (*n* = 696) cases from TCGA and GEO cohorts were combined and randomly divided into training (*n* = 348) and validation cohorts (*n* = 348) at a 1:1 ratio. The training cohort was used for subsequent analysis to construct the NRG_score. After univariate Cox regression analysis, Lasso regression was performed using the R package “glmnet” and multivariate Cox regression was eventually conducted to identify candidate prognostic necroptosis genes. The NRG_score was calculated using the formula: NRG_score = Σ (Exp * coefi). Patients were further divided into high-risk and low-risk groups based on the median NRG_score.

### Correlation of prognostic NRG risk score with clinical factors

Patients in the validation and all other cohorts were also divided into high-risk and low-risk groups using the same criteria. The distribution plot revealed a correlation between overall survival status and NRG_score. Kaplan–Meier analysis was performed to compare the prognosis of the different risk groups in overall survival (OS) among the training, validation, and all patient cohort. A heatmap was plotted to compare the differential expression patterns of prognostic NRGs between the two groups.

### Immune landscape, microsatellite instability status, cancer stem cell index, tumor mutation burden score, and HLA gene expression between high- and low-risk groups

The CIBERSORT algorithm was applied to explore the correlations of 22 infiltrating human immune cell types and NRG_scores. The ESTIMATE algorithm was performed to assess the immune and stromal scores between high-risk and low-risk groups. Correlations of expression of 12 prognostic NRGs and immune cells were additionally evaluated. We further examined the associations of the NRG_score and immune checkpoint gene expression, MSI status, CSC index, TMB score, and HLA gene expression. A mutation annotation format (MAF) was performed for comparison of GC patients in high-risk and low-risk groups using the maftools R package.

### Drug susceptibility analysis and immunotherapy response prediction

To explore the application value of NRG_score in clinical drug selection, “pRRophetic” R package analysis was performed to assess drug susceptibility in the two risk groups and the half-maximal inhibitory concentration (IC50) values calculated for commonly used chemotherapy or targeted therapeutic drugs. The Cancer Immunome Atlas (https://tcia.at/) analyzed the immune landscapes and antigenomes of 20 solid tumors that were quantified by Immunophenoscore (IPS, a superior immune response molecular marker). The IPS value, which ranged from 0 to 10, was positively correlated to tumor immunogenicity and could predict the patients’ response to immune checkpoint inhibitors therapy, including anti-PD-1/PD-L1 and anti-CTLA-4 immunotherapy. Imvigor210 was a transcriptome database including treatment response data of patients who received anti-PD-L1 immunotherapy. It was extracted to assess the predicting value of NRG risk score in immunotherapy response. Another public cohort GSE78220, was also used to assess the response and survival outcomes for patients receiving immunotherapy based on NRG risk score.

### Construction and validation of an NRG-related nomogram

A prognostic nomogram was constructed by integrating the NRG risk level with common clinical variables. We additionally generated calibration curves for 1-, 3-, and 5-year OS to compare the model prediction values with actual outcomes. Decision curve analysis (DCA) analysis was conducted to estimate the predictive value of the nomogram in clinical decision-making practice. A receiver operating characteristic (ROC) curve was plotted to compare the prognostic power of the NRG_score risk group alone with the nomogram model.

### Human tissues and quantitative real time-polymerase chain reaction

We obtained 16 cancer and their paired normal tissues from gastric cancer patients who underwent stomach surgery in the First Affiliated Hospital of Zhengzhou University. The study protocol was approved by the First Affiliated Hospital of Zhengzhou University Research Ethics Committee. Written informed consent was provided by each patient. Total RNA was extracted by Trizol reagent (Takara, Beijing, China) according to the manufacturer’s protocol. The synthesis of cDNAs corresponding to the mRNAs of interest depended on PrimeScript RT reagent Kit with gDNA Eraser (Takara) and SYBR Green Premix (Cowin Biosciences, Jiangsu province, China) with specific PCR primers (Sangon Biotech Co., Ltd, Shanghai, China). The data were normalized with GAPDH. The primers used in PCR assays were listed in Supplementary Table S15.

### Statistical analysis

Statistical analyses were performed using R software version 4.1.1 (2021-08-10). Data were considered statistically significant at *p*-values<0.05. All R scripts have been added in the Supplementary Material.

## Results

### Mutation landscape and copy number alterations of NRGs in GC

**FIGURE 2:**
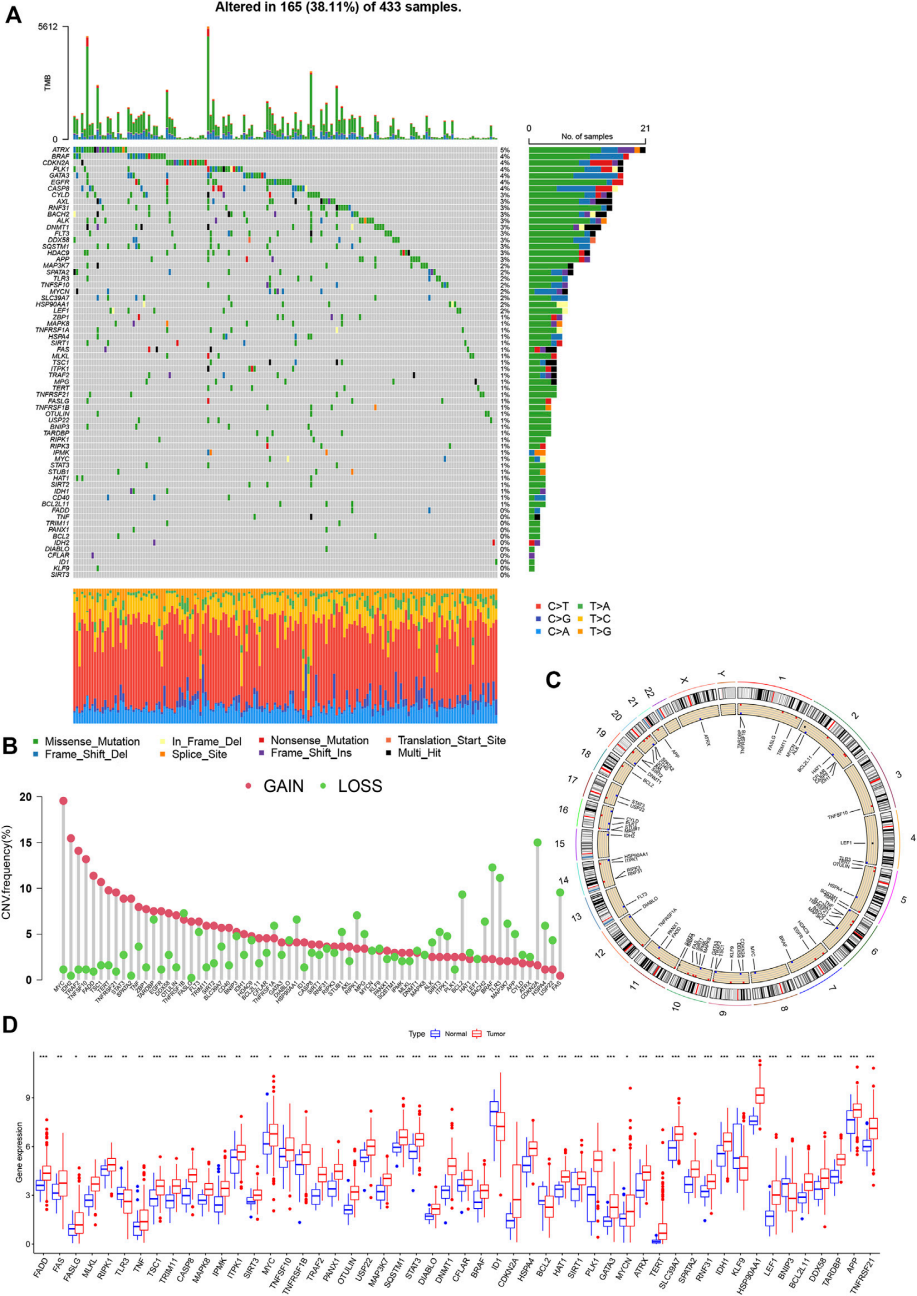
Genetic and transcriptional analysis of NRGs in GC. **(A)** Mutation frequencies of 67 NRGs in GC patients from TCGA cohort. **(B)** Copy number cariation of NRGs. **(C)** Locations of NRGs CNV on 23 chromosomes. **(D)** Expression differences of NRGs between normal and GC samples.

A total of 67 necroptosis-related genes (NRG) were included for analysis. Analysis of mutations revealed relatively high mutational frequencies of NRGs in GC. Among the 465 GC samples, 165 (38.11%) displayed NRG mutations (Figure 2A). Overall, 56 (83.6%) genes showed different mutation frequencies and types. The gene with the highest mutational frequency was *ATRX* (5%), followed by *BRAF*, *CDKN2A*, *PLK1*, *GATA3*, *EGFR*, and *CASP8* (4%). No mutations were detected in 11 NRGs (*SIRT3*, *KLF9*, *ID1*, *CFLAR*, *DIABLO*, *IDH2*, *BCL2*, *PANX1*, *TRIM11*, *TNF*, and *FADD*). Assessment of copy number variations (CNV) disclosed different CNV frequencies of all NRGs. *MYC, IDH2*, *TRAF2*, *TNFSF10*, and *FADD* exhibited a significant increase in CNV while CNV was decreased in *CDKN2A, BRAF, TLR3, FAS,* and *RIPK1* (Figure 2B). The locations of 67 NRGs in the 23 chromosomes were further investigated. Chromosome 2 was the most common location, housing *MYCN*, *ALK*, *HAT1*, *CFLAR*, *CASP8*, and *IDH1* (Figure 2C).

The mRNA levels were different between GC and normal samples for the majority of NRGs (Figure 2D). Expression patterns of mRNA were consistent with CNV results. For instance, *MYC*, *FADD*, *TERT*, and *TNFSF10* showed high expression in GC samples with CNV gain status while *TLR3*, *KLF9*, and *BCL2* were expressed at low levels in GC samples with CNV loss status. Some NRGs showed upregulated mRNA expression with CNV loss, such as *CDKN2A* and *RIPK1*, while the *ID1* gene with CNV gain was downregulated. These findings suggest transcriptional regulation by other potential mechanisms in addition to CNV. The observed background of NRG gene expression and mutation in addition to CNV and chromosomal localization data in GC patients support an important role of NRGs in the oncogenesis of gastric cancer.

### Necroptosis cluster identification in GC

**FIGURE 3:**
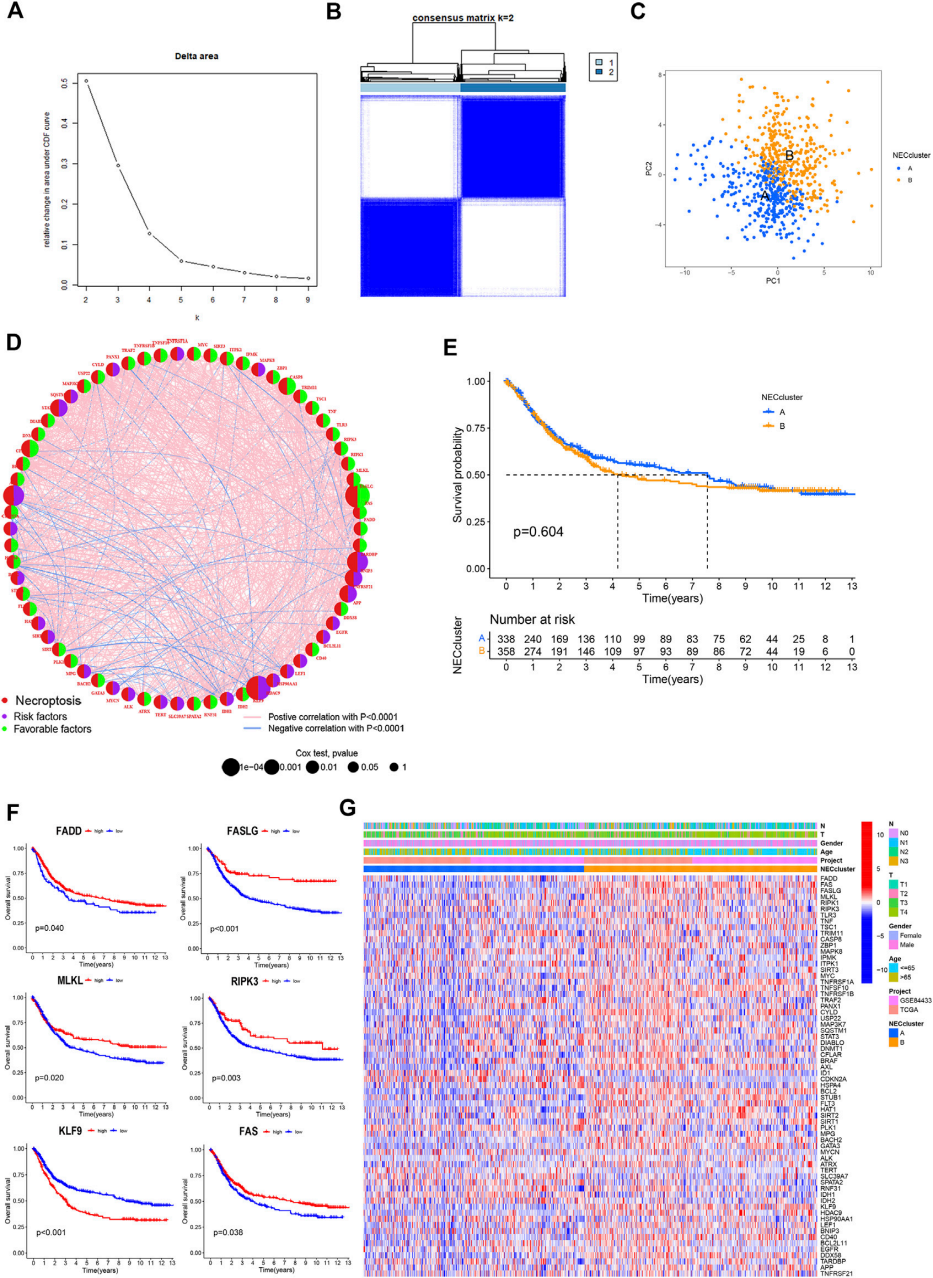
Necroptosis subtypes in GC. **(A,B)** Consensus matrix heatmap identifying 2 clusters (k = 2). **(C)** PCA analysis showing an obvious difference in necroptosis transcription profile. **(D)** Interactions among NRGs in GC. The line connecting the NRGs represents their interaction. Blue represents negative and pink represents positive correlations, respectively. **(E)** Survival analysis of NRG cluster according to OS in GC. **(F)** Kaplan-Meier curve showed that high expression of *PADD, FASLG, MLKL, RIPK3* and *FAS* were associated with better outcomes, while low expression of *KLF9* had a longer OS in GC. **(G)** Differences of the clinicopathological factors and NRGs expression between two necroptosis clusters.

Detailed information on GC patients from the TCGA and GSE84433 cohorts is presented in Supplementary Tables S2, S3. According to expression patterns of the 67 NRGs, GC patients were subdivided into different clusters using a consensus clustering algorithm (Supplementary Figure S1). The data indicate that k = 2 is an appropriate choice for the classification of patients into A (n = 340) and B (n = 360) subtypes (Figures 3A,B; Supplementary Table S4). At k = 2, the cumulative distribution function curve (CDF) increased gradually, and the sample size was relatively balanced between the two subgroups. In principal component analysis (PCA), subtypes A and B showed obvious differences in necroptosis transcription profiles (Figure 3C). Univariate Cox regression analysis was applied to determine the prognostic significance of the 67 NRGs in GC (Supplementary Table S5). Based on univariate Cox regression results, a necroptosis network was generated showing the interrelationship between each necroptosis gene and their predictive value in GC (Figure 3D). The Kaplan–Meier curve showed a relatively longer OS in subtype_A than subtype_B patients, but these differences were not significant (*p* = 0.604, Figure 3E). Most of the necroptosis genes were associated with the prognosis of gastric cancer patients. Kaplan–Meier survival analysis revealed an association of high expression of *PADD*, *FASLG*, *MLKL*, *RIPK3*, and *FAS* with better outcomes while low expression of *KLF9* was correlated with longer OS in GC (Figure 3F). Heatmap was performed to reveal relative differences in NRG expression and clinical features between the two subgroups (Figure 3G).

### Identification and functional analysis of DEGs

**FIGURE 4:**
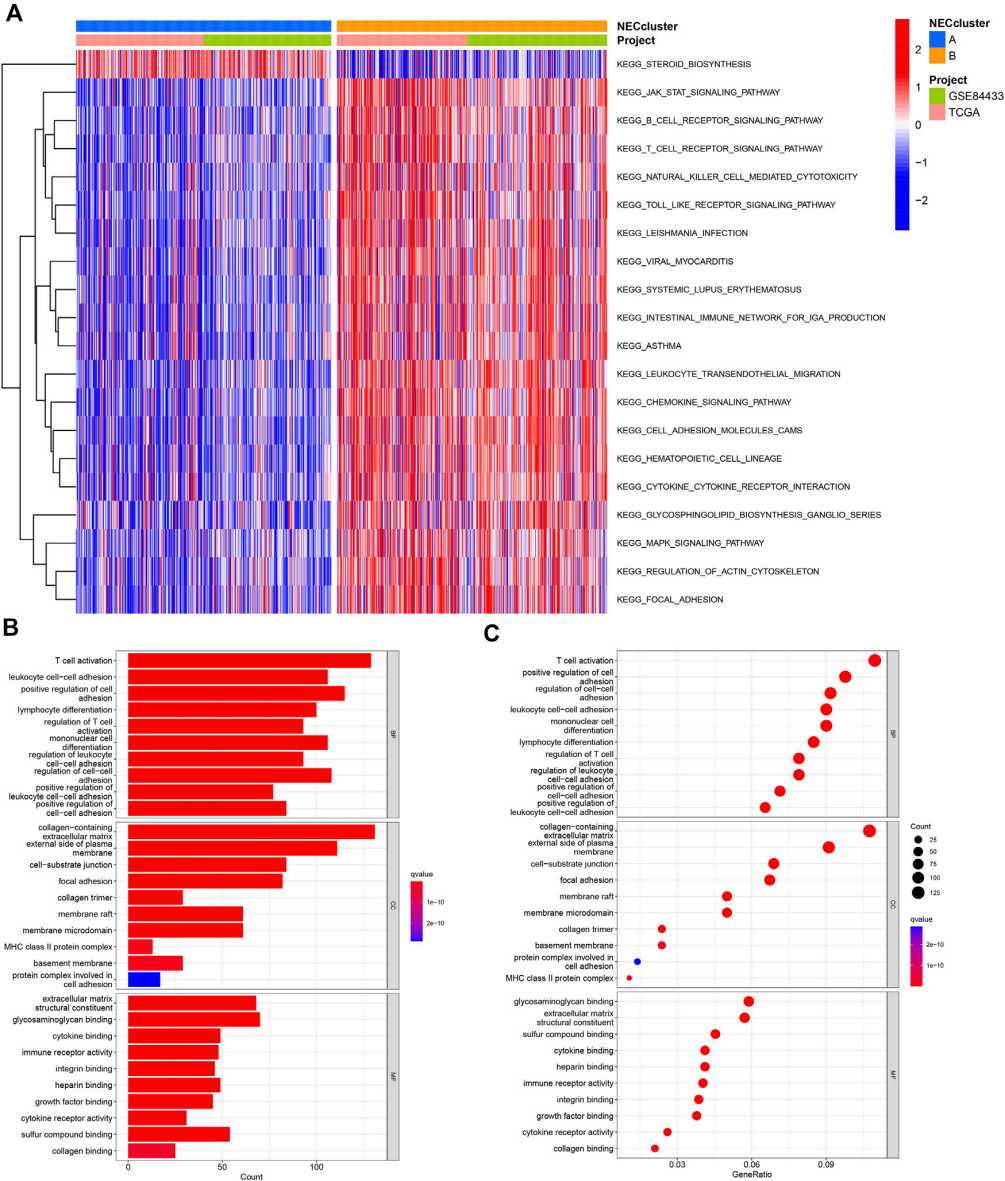
Functional analysis of necroptosis subtypes in GC. **(A)** CSVA analysis of the biological pathways between necroptosis subtypes. **(B,C)** GO and KEGG analysis of DEGs between two necroptosis subtypes.

To determine potential functional differences between the two necroptosis subgroups, we performed GSVA analysis, which showed significant enrichment of subtype_A in the steroid biosynthesis pathway (Figure 4A, Supplementary Table S6) and enrichment of subtype_B in essential immune-related pathways, including JAK-STAT signaling, B cell receptor signaling, T cell receptor signaling, natural killing cell-mediated cytotoxicity, and leukocyte transendothelial migration.

We identified 1239 necroptosis-related DEGs between subtypes A and B using the “limma” R package (Supplementary Table S7). Functional GO analysis revealed that these DEGs were mainly enriched in biological processes associated with tumor immunity, such as T cell activation, lymphocyte differentiation, regulation of T cell activation, and cell-cell adhesion, indicative of an important role of necroptosis in the tumor microenvironment (Figures 4B,C; Supplementary Table S8).

### Necroptosis gene cluster based on prognostic DEGs

**FIGURE 5:**
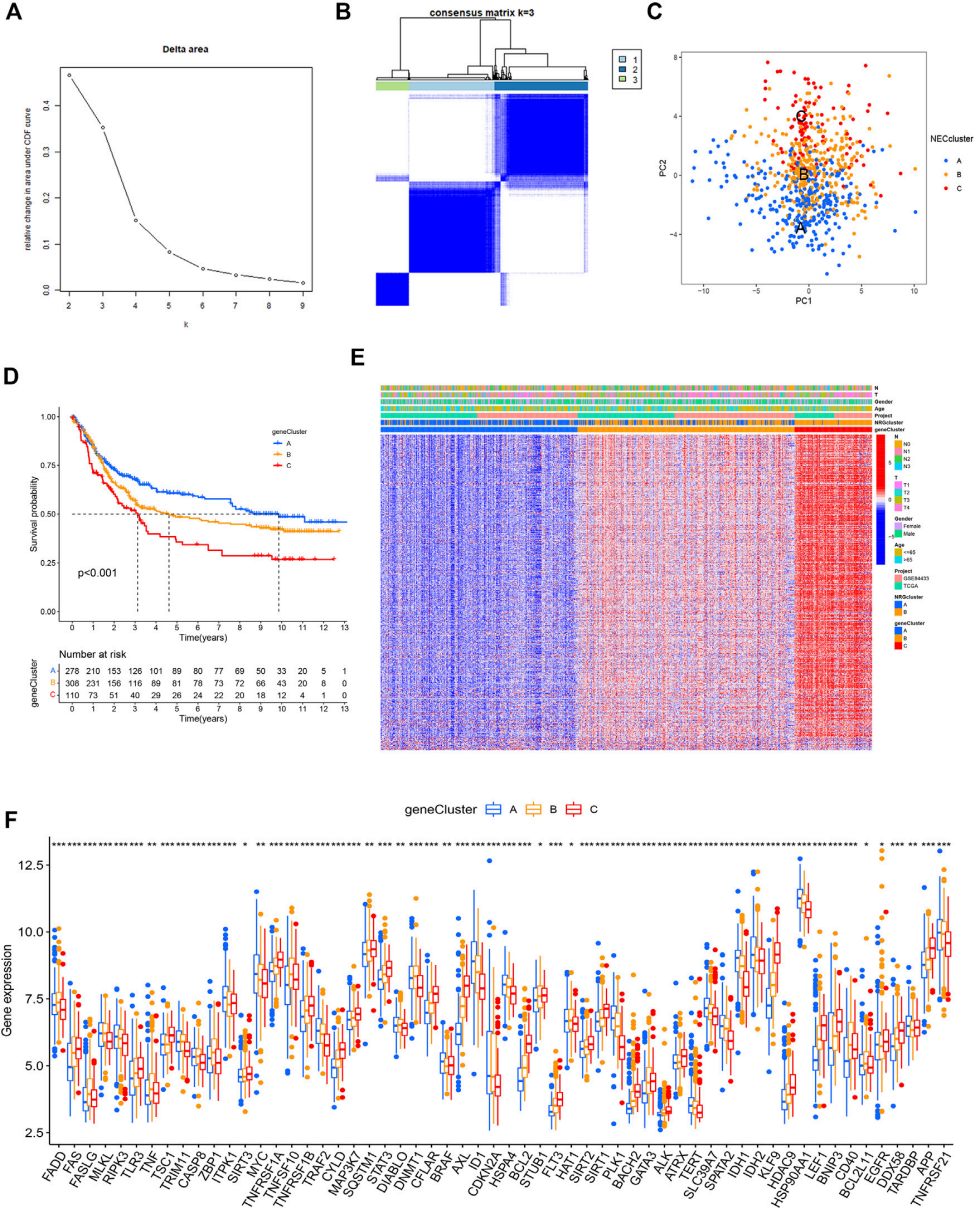
Gene clusters based on prognostic DEGs. **(A,B)** Consensus matrix heatmap identifying 3 clusters (k = 3). **(C)** PCA analysis showing an obvious difference in necroptosis transcription profile. **(D)** Survival analysis showing gene clusters were associated with OS in GC patients. **(E)** Differences of the clinicopathological factors and NRGs expression between three gene clusters. **(F)** Differences of NRGs expression between three gene clusters.

Univariate Cox regression analysis was performed based on 1239 DEGs and among which 606 genes related to OS for GC were selected (Supplementary Table S9). A new consensus clustering was conducted based on these prognostic NRGs and GC patients were divided into three necroptosis gene subtypes designated gene subtype_A, gene subtype_B, and gene subtype_C (k = 3, Figures 5A,B; Supplementary Figure S2; Supplemntary Table S10). PCA showed significant differences in the necroptosis transcription profiles of subtypes A, B, and C (Figure 5C). In Kaplan–Meier analysis, gene subtype_A had the highest OS while the shortest outcome was determined for subtype_C (*p* < 0.001, Figure 5D). The relative differences in T, N classification, gender, age, and necroptosis clustering between A, B, and C gene subtypes are presented in Figure 5E. In addition, significant differences in NRG expression among the three gene subtypes were observed (Figure 5F).

### Prognostic NRG risk score

**FIGURE 6:**
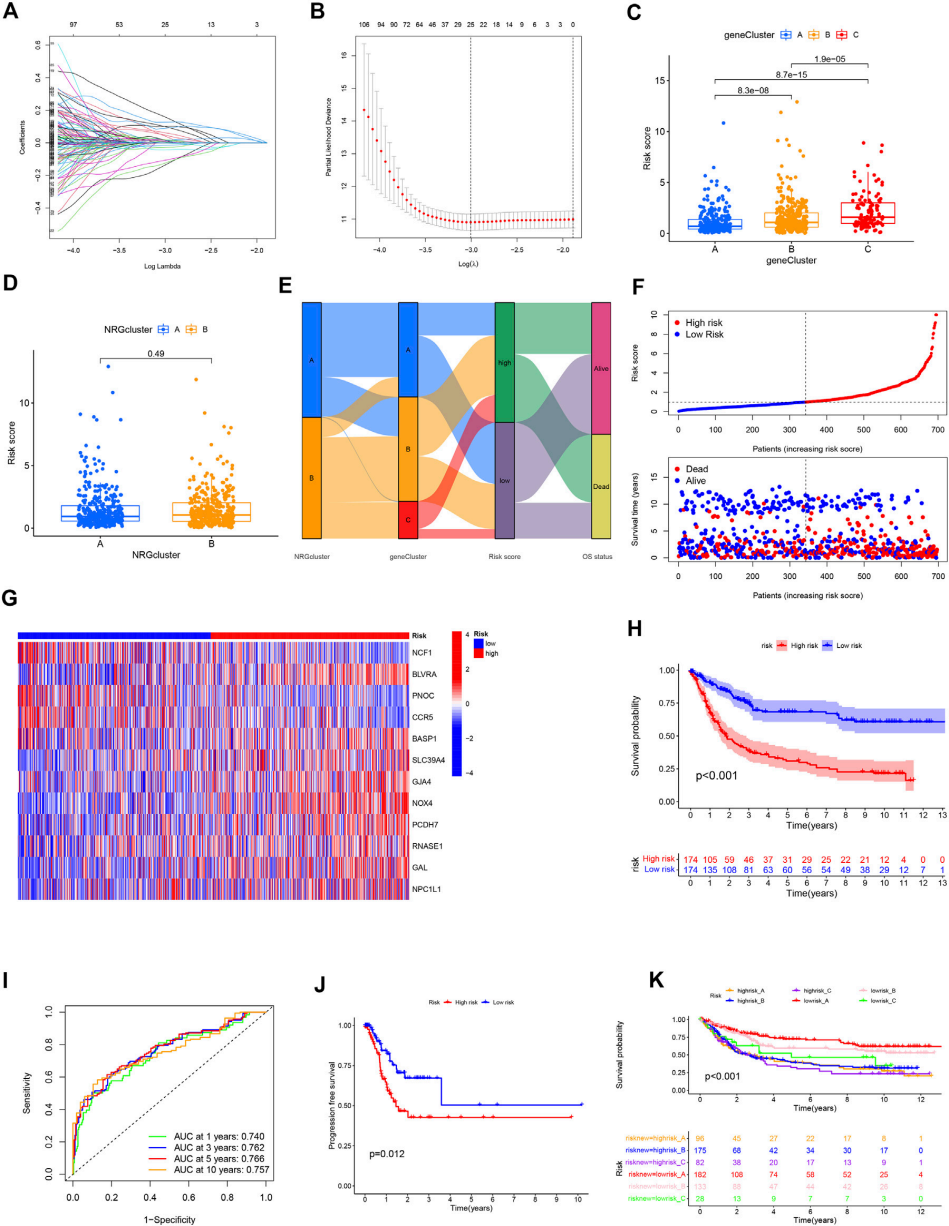
Construction of the NRG risk score in GC patients. **(A,B)** The LASSO regression analysis and partial likelihood deviance on the prognostic genes. **(C,D)** NRG risk score distribution in necroptosis and gene subtypes. **(E)** Correlation of necroptosis subtype, gene subtype, NRG risk score and survival outcomes. **(F)** Ranked dot and scatter plot showing the NRG risk score and survival status in all GC patient cohort. **(G)** The 12 prognostic NRG genes in the model between high and low-risk groups. **(H)** Survival analysis of the OS between the high and low-risk groups. **(I)** ROC analysis of the predictive ability of 1,3,5 and 10-year OS according to NRG risk score. **(J)** Survival analysis of the PFS between the high and low-risk groups. **(K)** Survival analysis according to the gene cluster and NRG risk level.

GC patients from TCGA and GEO cohorts were combined and randomly divided into training and validation cohorts at a 1:1 ratio. The training cohort was used for subsequent analyses. LASSO analysis was performed for 606 prognostic DEGs and 25 genes selected for subsequent multivariate Cox regression analysis (Figures 6A,B). Finally, 12 prognostic NRGs were identified (Supplementary Table S11), including 9 high-risk genes (*NPC1L1*, *GAL*, *RNASE1*, *PCDH7*, *NOX4*, *GJA4*, *SLC39A4*, *BASP1*, and *BLVRA*) and 3 low-risk genes (*NCF1*, *PNOC*, and *CCR5*). Correlation of the 12 model genes expression with OS in the whole patients set was also presented in Supplementary Figure S3. Based on the results of multivariate Cox regression analysis, the NRG risk score was calculated as follows:

NRG risk score = (–0.43594* expression of *NFC1*) + (–0.38714* expression of *PNOC*) + (–0.27229* expression of *CCR5*) + (0.46847* expression of *BLVRA*) + (0.29272* expression of *BASP1*) + (0.28869* expression of *SLC39A4*) + (0.28408* expression of *GJA4*) + (0.24301* expression of *NOX4*) + (0.18931* expression of *PCDH7*) + (0.18843* expression of *RNASE1*) + (0.09485* expression of *GAL*) + (0.07200* expression of *NPC1L1*).

Based on the median NRG risk score, all GC patients were divided into high-risk and low-risk groups. The majority of necroptosis-related genes were differentially expressed between the two risk groups (Supplementary Figure S4). Correlations between risk level with clinicopathological factors were presented in Table1. It showed that high NRG risk score was positively related with advanced T stage, N stage and gene cluster C subtype. NRG risk score was significantly different among the three gene clusters. The NRG score was highest in gene cluster_C and lowest in gene cluster_A (Figure 6C) while no significant differences were observed between necroptosis clusters A and B (Figure 6D).

**TABLE1:** Patient Characteristics according to necroptosis risk score.

Characteristics	High risk N = 353	Low risk N = 342	*p*-value*
Age,years			0.413
Median (IQR)	64.0 (54, 70)	63.5 (56, 70)	
Range	30, 90	27, 90	
Gender			0.811
Female	124 (35%)	117 (34%)	
Male	229 (65%)	225 (66%)	
Grade			0.981
G1	4 (1.1%)	4 (1.2%)	
G2	61 (17%)	63 (18%)	
G3	102 (29%)	97 (28%)	
missing	186 (53%)	178 (52%)	
Stage			0.186
Stage I	18 (5.1%)	28 (8.2%)	
Stage II	54 (15%)	48 (14%)	
Stage III	63 (18%)	71 (21%)	
Stage IV	22 (6.2%)	12 (3.5%)	
missing	196 (56%)	183 (54%)	
T			0.006
T1	9 (2.5%)	19 (5.6%)	
T2	51 (14%)	56 (16%)	
T3	100 (28%)	123 (36%)	
T4	187 (53%)	142 (42%)	
missing	6 (1.7%)	2 (0.6%)	
N			0.002
N0	65 (18%)	104 (30%)	
N1	125 (35%)	119 (35%)	
N2	100 (28%)	69 (20%)	
N3	55 (16%)	42 (12%)	
missing	8 (2.3%)	8 (2.3%)	
M			0.159
M0	147 (42%)	154 (45%)	
M1	16 (4.5%)	7 (2.0%)	
missing	190 (54%)	181 (53%)	
NECcluster			0.081
A	160 (45%)	178 (52%)	
B	193 (55%)	164 (48%)	
geneCluster			<0.001
A	96 (27%)	181 (53%)	
B	175 (50%)	133 (39%)	
C	82 (23%)	28 (8.2%)	

*Welch Two Sample t-test; Fisher’s exact test.

The Sankey plot allowed visualization of the interrelationships among two necroptosis clusters, three gene clusters, risk_score, and overall survival status (Figure 6E). The distribution plot showed that OS of GC decreased with increased NRG risk score (Figure 6F). For all patient cohorts, a heatmap was generated to establish the relationships of the 12 prognostic marker genes with NRG risk groups. *BLVRA*, *SLC39A4*, *GJA4*, *NOX4*, *PCDH7*, and *GAL* were highly expressed in the high-risk group while *NCF1*, *PNOC*, and *CCR5* were highly expressed in the low-risk group (Figure 6G). The correlation results in the training and validation sets are presented in Supplementary Figure S5. Kaplan–Meier curve analysis showed significantly longer OS in the low-risk relative to the high-risk group (*p* < 0.001, Figure 6H). ROC curves indicated that NRG risk score had promissing value in predicting OS of GC patients, with AUC at 1, 3, 5, and 10 years of 0.740, 0.762, 0.766, and 0.757, respectively (Figure 6I). Progression free survival (PFS) was also higher in low-risk than high-risk group GC patients (Figure 6J). We obtained similar results with the whole patient and validation sets (Supplementary Figures S6, S7). Risk group and gene cluster data were combined for the subclassification of GC patients into six different subgroups. The lowrisk_A group had better OS relative to the other subgroups. Low-risk groups could be further divided into three survival outcome groups while the high-risk group consistently showed the poorest survival rates within our patient population (Figure 6K).

### Validation of the expression levels of the 12 NRGs in the prognostic model

The expression levels of 12 prognostic genes were measured in 16 GC tissues and 16 paired adjacent normal tissues by RT-qPCR. As shown in Supplementary Figure S8, all of the 12 NRGs were significantly differentially expressed between cancer and normal tissues. The expression of *PNOC* and *RNASE1* were downregulated and other genes (*BASP1*, *BLVRA*, *CCR5*, *GAL*, *GJA4*, *NCF1*, *NOX4*, *NPC1L1*, *PCDH7*, and *SLC39A4*) were upregulated in GC tissues than corresponding normal tissues. Results of RT-qPCR were presented in Supplementary Table S16.

### Tumor microenvironment in the high- and low-risk groups

**FIGURE 7:**
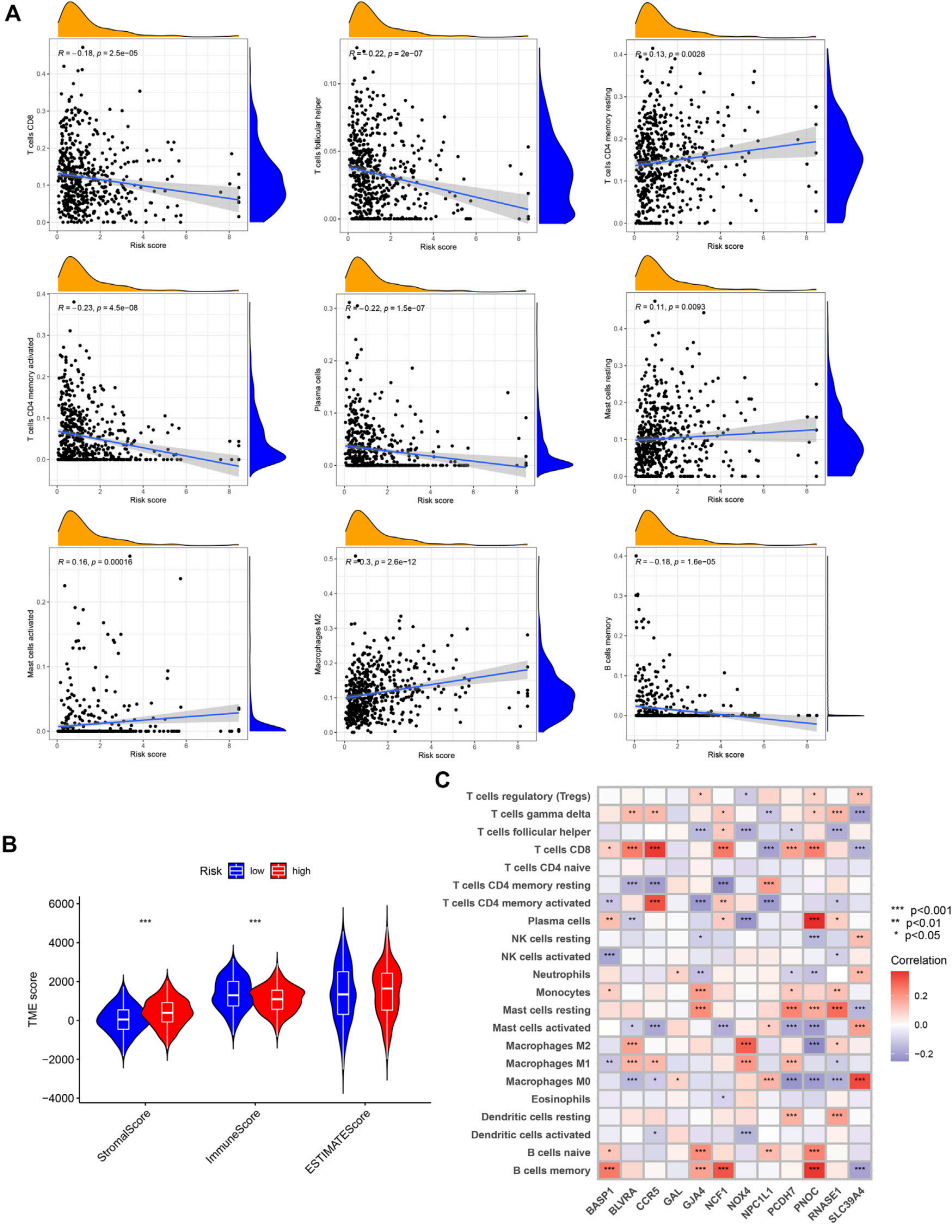
Association of TIME with risk_score. **(A)** Correlation of infiltrating immune cells with NRG risk score. **(B)** Correlation of NRG risk score with immune/stromal scores. **(C)** Correlation of immune cells with 12 NRGs.

**FIGURE 8:**
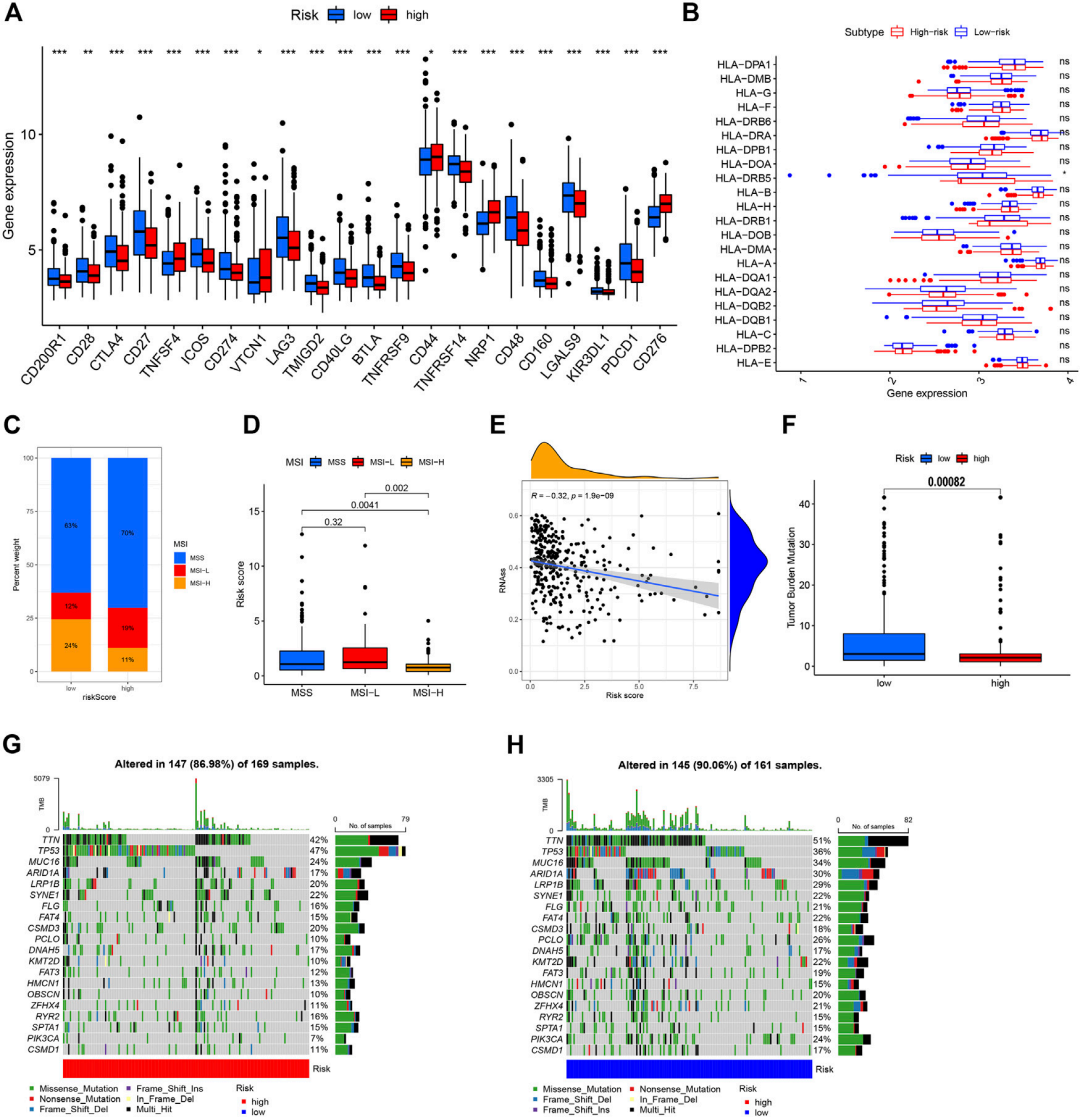
Comprehensive analysis of NRG risk score in GC. **(A)** Correlation of NRG risk score and immune checkpoint gene expression. **(B)** Correlation of NRG risk score and HLA gene expression. **(C,D)** Correlation of NRG risk score and MSI status. **(E)** Correlation of NRG risk score and CSC index. **(F)** Correlation of NRG risk score and TMB. **(G,H)** Somatic mutation features in the high- and low-risk groups.

CIBERSORT algorithm results revealed positive associations of the prognostic NRG risk score with resting CD4 memory cells, resting mast cells, activated mast cells, and M2 macrophages, and negative correlations with CD8^+^T cells, follicular helper T cells, activated memory CD4^+^T cells, plasma cells, and memory B cells (Figure 7A, Supplementary Table S12). The ESTIMATE algorithm was additionally performed to simulate TIME. The results showed that high NRG risk score was associated with low immune score and high stromal score in GC samples (Figure 7B, Supplementary Table S13), suggesting that the high-risk group has a relatively good immune microenvironment. The relationship between 12 prognostic necroptosis genes and 22 human immune-related cells was further examined (Figure 7C). The majority of immune cells were significantly positively or negatively regulated with the 12 genes. The relationship between NRG prognostic risk_score and immune checkpoints was further assessed (Figure 8A). Analysis of 33 immune checkpoint genes led to the identification of 22 that were differentially expressed between the two risk groups.

### Correlations of NRG risk score with MSI, CSC index, HLA gene expression, and TMB score

Experiments were performed to determine the relationship of NRG risk score with immunotherapeutic biomarkers, such as MSI, CSC index, HLA gene expression, and TMB score. Notably, a low NRG risk score was significantly correlated with MSI-H status. In the low-risk group, MSI-H status was 24%, which was higher than that in the high-risk group (Figure 8C), and the median risk_score of MSI-H was significantly lower than that of MSI-L/MSS groups (Figure 8D). The NRG risk score showed a negative linear correlation with the CSC index (R = –0.32, *p* < 0.001, Figure 8E), indicating that GC cells with high NRG risk score have less distinct stem cell properties and a higher level of cell differentiation. We additionally performed a correlation analysis between NRG risk score and HLA gene expression. Our results showed lower expression of *HLA-DRB5* in the high-risk relative to the low-risk group (Figure 8B, Supplementary Table S14). The TMB score was negatively associated with the NRG gene cluster in this study (Supplementary Figure S9) and significantly lower in the high-risk than the low-risk group (Figure 8F). Next, we analyzed the distribution of somatic mutations between the two necroptosis risk groups in the TCGA GC cohort. Low-risk samples showed relatively higher mutation frequency than the high-risk group (90.06% vs. 86.98%, Figures 7G,H). Multiple genes displayed mutations in gastric cancer cells but the types and frequencies were distinct. For example, the frequency of *TTN* mutation was 47% in the high-risk group and 36% in the low-risk group. The top 5 mutated genes in the high-risk group were *TP53*, *TTN*, *MUC16*, *SYNE1*, and *LRP1B*, while *TTN*, *TP53*, *MUC16*, *ARID1A*, and *LRP1B* were the top 5 genes showing mutations in the low-risk group.

### Drug susceptibility analysis

**FIGURE 9:**
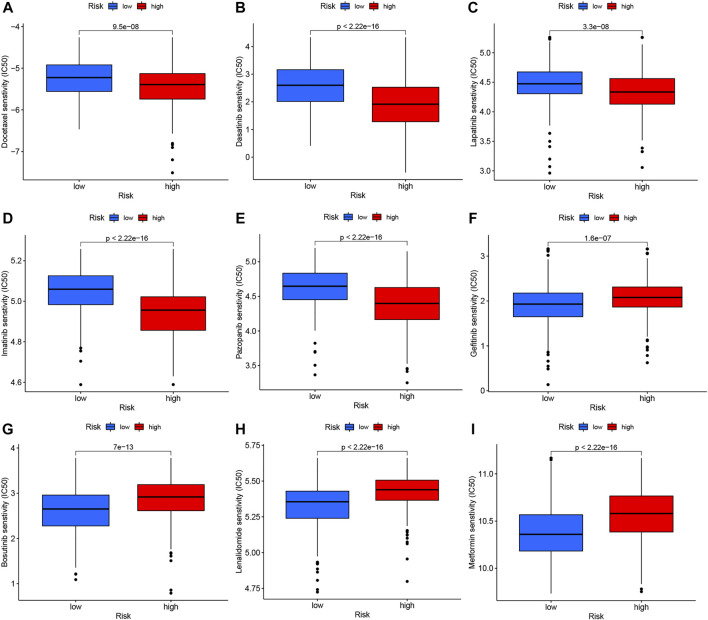
Drug susceptibility analysis of chemotherapy and targeted therapy in high- and low-risk groups. **(A)** Docetaxel, **(B)** Dasatinib, **(C)** Lapatinib, **(D)** Imatinib, **(E)** Pazopanib, **(F)** Gefitinib, **(G)** Bosutinib, **(H)** Lenalidomide, and **(I)** Metformin.

We performed drug susceptibility analysis to select promising chemotherapy or targeted drugs for high- and low-risk groups of GC. Interestingly, patients in the high-risk group had lower IC_50_ values for docetaxel, lapatinib, pazopanib, dasatinib, and imatinib (Figures 9A–E) while those in the low-risk group had significantly lower IC_50_ values for gefitinib, metformin, bosutinib, lenalidomide, and salubrinal (Figures 9F–I). These results support the utility of NRG risk score in the prediction of drug sensitivity and selection of potential beneficiaries of specific treatment drugs among GC patients.

### NRG risk score predicts response to immunotherapy

**FIGURE 10:**
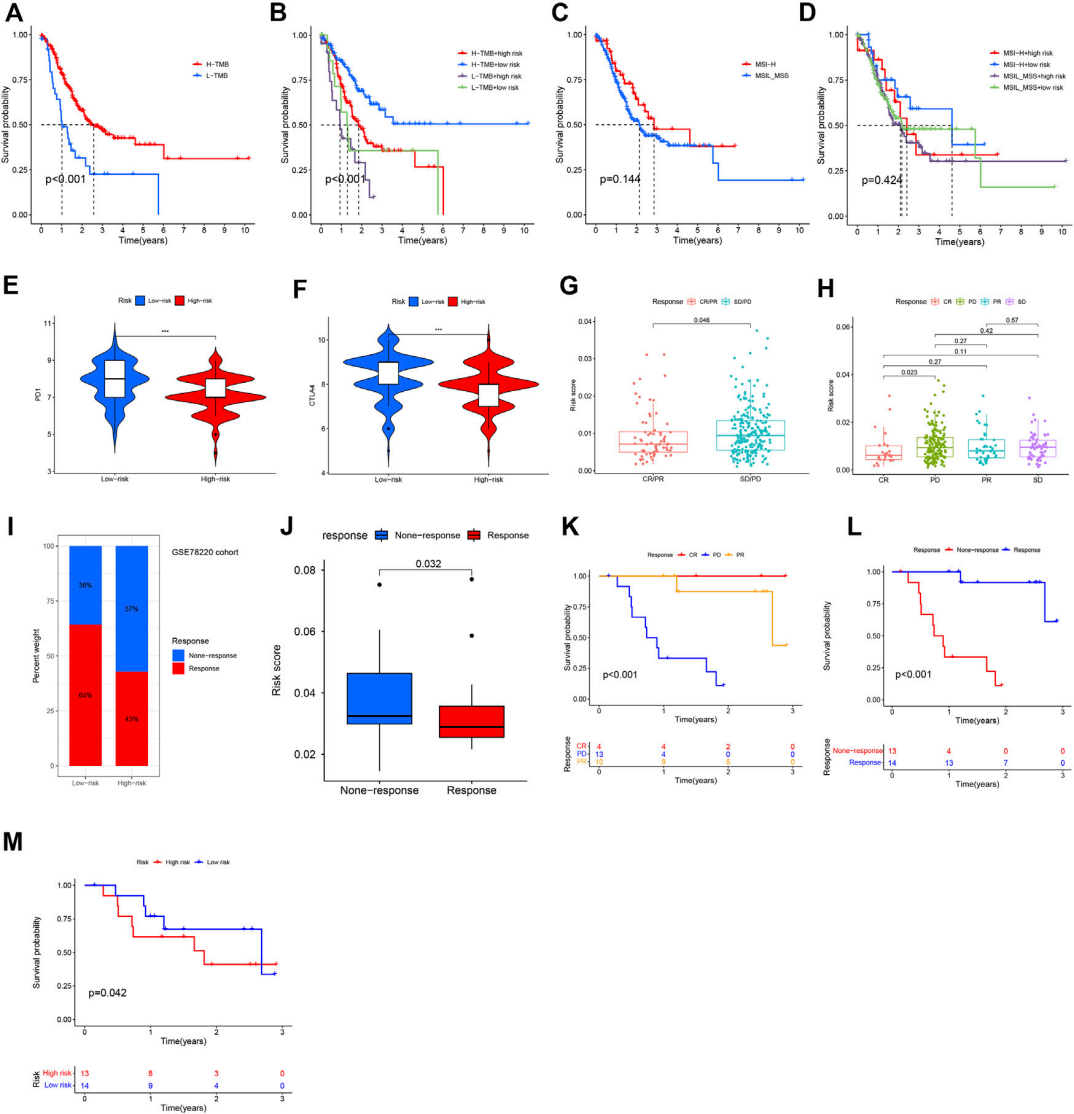
Immunotherapy response based on necroptosis risk score. **(A–D)** K-M analysis based on TMB, MSI status, or their combination with NRG risk score. **(E,F)** Predicting of response to anti-CTLA-4 and anti-PD-1/PD-L1 therapy (IPS score) in the low-risk score and high-risk score groups in TCGA-GC patients in this study. **(G,H)** Correlation of immunotherapy response with NRG risk score according to IMvigor210 database. **(I,J)** Correlation of NRG risk score with ICIs response. **(K,L)** Survival in CSE78220 cohort related to ICIs response. **(M)** Survival in CSE78220 cohort related to NRG risk score.

As the important biomarker for immunotherapy, we firstly performed K-M analysis based on TMB and MSI status in GC patients. It showed that High-TMB patients had a significantly better OS than low-TMB GC patients (Figure 10A **,** *p* < 0.001) and when combining with risk levels (Figure 10B). While, there was no difference between MSI-H and MSI-L/MSS patients (Figure 10C, *p* = 0.144), even combining with risk levels (Figure 10D, *p* = 0.424).

According to results of TCIA database analysis, IPS scores were download and correlation with NRG risk score was also evaluated. IPS-PD-1/PD-L1/PD-L2_pos and IPS-CTLA-4_pos was higher in low-risk group patients than in high-risk group patients (Figures 10E,F), indicating that low-NRG_risk gastric cancer patients might have higher tumor immunogenicity and benefit from anti- PD-1/PD-L1/PD-L2 and anti- CTLA-4 blocker immunotherapy. In Imvigor210 cohort, we also found that NRG risk score was significantly correlated with patients’ objective response of immunotherapy. Risk score in the response group (CR/PR, complete response and partial response) group was obviously lower than none-response group (SD/PD, stable disease and progressive disease, *p* = 0.046, Figure 10G), expecially between CR and PD groups patients (Figure 10H, *p* = 0.023), indicating that NRG risk score could act as a potentially promising therapeutic biomarker of immunotherapy in gastric cancer.

Next, we evaluated the prognostic value of NRG risk score in a melanoma treated by anti-PD-1 cohort (GSE78220). Percentage of response to ICIs in the low-risk group was higher than high-risk group (Figure 10I), and the median NRG risk score in response group patients was significantly lower than none-response group patients (*p* = 0.038, Figure 10J). K-M analysis showed that patients with a response to ICIs (CR and PR) had a better OS than none-response (PD) group patients (Figures 10K,L, *p* < 0.001). At last, we assess the NRG risk score in survival of ICIs treated patients and the promising result showed that it was significantly correlated with OS in this study (*p* = 0.042, Figure 10M), indicating that NRG risk score might be a useful biomarker for immunotherapy.

### Construction and validation of nomogram based on NRG risk score

**FIGURE 11:**
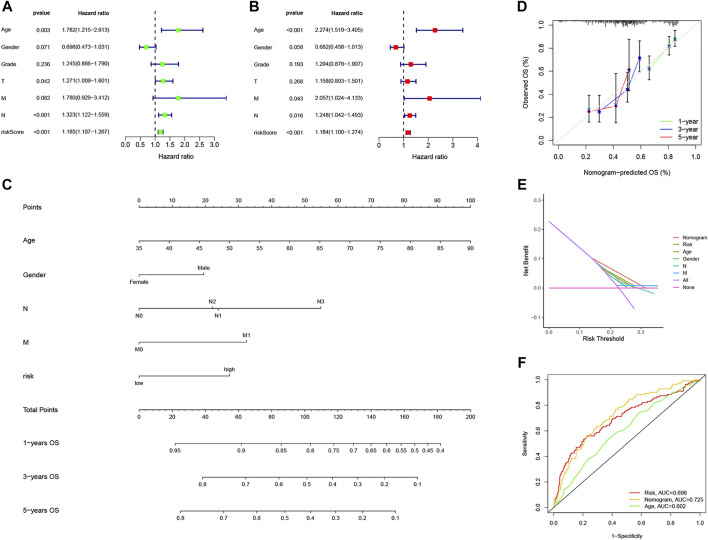
Construction and validation of the nomogram based on NRG risk score. **(A,B)** Forest plots of univariate and multivariate Cox Regression analysis. **(C)** Nomogram predicting 1-, 3-, and 5-year OS in GC patients. **(D)** Calibration curve for prediction of 1-,3- and 5-year OS. **(E)** Decision curve analyses of the nomogram. **(F)** ROC curves for predicting OS in GC patients.

We integrate NRG risk score and the common clinicalpathological factors, including age, gender, T stage, N stage, M stage and pathological grade, and performed univariate Cox regression analysis and subsequent multivariate Cox regression analysis. It revealed that NRG risk score was an independent prognostic factor for GC patients in this study (*p* < 0.001, Figures 11A,B). Factors showed significance in the multivariate analysis were incorporated into a nomogram. For gender acts as an easy and common factor in clinical practice, we also consider it in the model construction (Figure 11C). The calibration curves of the nomogram indicated excellent consistency with the standard curve between predicted and actual 1-, 3-, and 5-year OS rates in GC patients (Figure 11D). DCA was conducted to evaluate the predictive value of the nomogram in clinical decision-making (Figure 11E). Notably, the nomogram showed better reliability than the common clinicopathological factors or NRG risk score alone. The AUC value of the nomogram was 0.725, which was higher than the NRG risk score (0.696, Figure 11F), indicating a better predictive value than NRG risk score alone.

## Discussion

Apoptosis is a common pathway of programmed cell death in the body. Traditional chemotherapeutic drugs mainly exert their effects by promoting apoptosis of tumor cells, with resistance to apoptosis identified as the predominant mechanism underlying tumor drug resistance (Mortezaee et al., 2019; Carneiro and El-Deiry, 2020). A comprehensive investigation of the pathways of tumor cell apoptosis has led to the gradual discovery of novel cells death modes, such as pyroptosis, ferroptosis, cuproptosis, and necroptosis. In contrast to apoptosis, necroptosis rarely presents with cytoplasmic shrinkage, chromatin condensation, nuclear fragmentation, cell membrane blebbing, or shedding of apoptotic bodies (Pasparakis and Vandenabeele, 2015; Su et al., 2016). Necroptosis is reported to play potential roles in several diseases including inflammatory bowel disease, multiple dermatosis, acute kidney injury, inflammatory response syndrome, and atherosclerosis. Necroptosis is also implicated in cancer, with reports of its involvement in oncogenesis, adaptive immunity, cancer subtype, progression, and metastasis. Evidence from several studies supports the protective role of necroptosis against tumor development (Seifert et al., 2016; Strilic et al., 2016).

Gastric cancer is a fatal disease with poor overall survival statistics worldwide. Several factors (including low rates of early diagnosis, high intratumor heterogeneity, and drug resistance) contribute to the poor survival outcomes of GC patients (Johnston and Beckman, 2019; Thrift and El-Serag, 2020). The majority of patients present with distant metastasis at the time of diagnosis and therefore undergo chemotherapy. However, due to dose-limiting toxicity and limited responses, patients commonly experience disease progression following chemotherapy. The development of targeted therapy for advanced gastric cancer focuses on two aspects: *HER-2* and antiangiogenesis. Targeted therapy for *HER-2* as a first-line treatment is reported to improve survival in GC patients with positive *HER-2* expression (Sawaki et al., 2012). Ramucirumab, an antagonist of vascular endothelial growth factor receptor 2 (*VEGFR2*), has been shown to inhibit ligand-induced VEGFR2 activation, thereby suppressing ligand-induced proliferation and migration of human endothelial cells. Ramucirumab has been approved as a second-line treatment for gastric cancer and gastroesophageal junction carcinoma (Wilke et al., 2014). Although several new drugs have been identified, the availability of appropriate targeted therapy remains limited to a small number of GC patients. The advent of immunotherapy offers new options, and promising survival outcomes have been obtained for some patients receiving immunotherapy combined with chemotherapy according to PD-L1 expression or MSI status. In clinical practice, the identification of patient subgroups that could potentially benefit from immunotherapy is critical for improving outcomes. The field of gastric cancer therapy thus faces great opportunities and challenges (Zhao et al., 2019; Xie et al., 2021).

This study investigated the genetic features and transcriptional alterations in necroptosis-related genes in GC. Previous studies have indicated that cancer cells eradicate necroptosis by downregulating NRGs to evade necroptosis-induced cell death mainly involving RIP1, RIP3, and MLKL. In GC, the majority of NRGs were expressed at higher levels in tumor than normal samples. An earlier report demonstrated the downregulation of MLKL, a key regulator of the necroptosis axis, in PDAC, which was associated with lower OS (Colbert et al., 2013). Our experiments showed higher expression of MLKL in GC than in normal samples. Moreover, lower expression was associated with better survival outcomes, suggesting that MLKL has a promising prognostic ability. However, expression patterns of NRGs may differ among tumor types. Based on NRG expression, GC samples were divided into two cluster subtypes (A and B). While differences in OS were not significant between the two groups, we observed an extension of median survival time in group A. Function of enrichment analysis revealed enrichment of several immune activation-related signals in cluster B, including B cell receptor signaling, T cell receptor signaling, NK cell-mediated cytotoxicity, and JAK-STAT signaling, supporting potential correlation of necroptosis-related cancer subtypes with the tumor immune microenvironment. These findings provide novel insights and directions for future research. We further identified 1239 necroptosis-related DEGs between subtypes A and B, among which 606 were proven to be associated with OS in GC patients. According to the 606 prognostic DEGs, patients were further divided into three necroptosis gene clusters (gene subtype_A, gene subtype_B, and gene subtype_C). The gene cluster system was highly associated with OS of GC patients and most NRGs were differentially expressed among the three gene clusters. The collective results support the potential of NRGs as prognostic and immunotherapeutic biomarkers.

Next, we constructed and validated a useful prognostic model based on NRG risk score. NRG risk scores between the two cluster systems were additionally examined, revealing that survival was not significantly different between clusters A and B but differed among the three gene clusters. Based on NRG risk score, patients were eventually classified into high- and low-risk groups. Patients in the low-risk group had significantly better OS than those in the high-risk group. We subsequently explored the tumor immune microenvironment between the high- and low-risk groups. Several human immune cells were correlated with NRG risk score, including resting CD4 memory cells, resting mast cells, activated mast cells, M2 macrophages, CD8^+^T cells, follicular helper T cells, and activated memory CD4^+^T cells, plasma cells, and memory B cells. Marked differences in the immune score, stromal score, MSI status, TMB score, CSC index, HLA gene expression, and immune checkpoint expression levels were recorded between high- and low-risk groups. Drug susceptibility analysis may be used in clinical practice to select potentially effective drugs for GC based on necroptosis gene expression. Here, the first prognostic nomogram based on necroptosis in GC patients was constructed and its predictive ability assessed.

Current treatments for gastric cancer include resection of the tumor via gastroscopy or surgery, chemotherapy, targeted therapy, and radiotherapy. Despite considerable progress in the development of treatment strategies for gastric cancer, therapeutic outcomes remain unsatisfactory. Immunotherapy has been documented as an effective strategy for various tumor types, including gastric cancer, due to its precise effects on the tumor microenvironment and persistent response. While immunotherapy offers new hope, many patients do not benefit from this mode of treatment, mainly due to tumor heterogeneity and complex tumor immune microenvironments, which should be a focus of further research. The TIME is extremely complex and includes surrounding blood vessels, immune cells, fibroblasts, bone marrow-derived inflammatory cells, various signaling molecules, and an extracellular matrix (Pitt et al., 2016; Cassim and Pouyssegur, 2019). The tumor microenvironment has been shown to play an important role in malignant progression, immune escape, and immunotherapy resistance by altering the ratio of immunosuppressive and cytotoxic responses in the vicinity of the tumor. Rather than forming apoptotic bodies during cell apoptosis, necroptosis is accompanied by rupture of the cell membrane and release of tumor neoantigens, which can trigger strong inflammatory and anti-tumor immune responses (Gong et al., 2019). Based on the necroptosis risk_score in this study, we showed that TIME and the majority of key immune cells were significantly different between high- and low-risk groups.

T cells, which are essential immune cells, play critical roles in antigen recognition, presentation, and tumor cell killing in gastric cancer (Thommen and Schumacher, 2018; Choi et al., 2020). In our experiments, CD8^+^ and activated memory CD4^+^ T cells were negatively correlated with NRG risk score, indicating a critical role of necroptosis in the anti-tumor immune response of gastric cancer. Limited research to date has focused on the anti-tumor role of B cells. Recent accumulating evidence suggests that B cells may serve as an important prognostic factor. B cells are active participants that fundamentally coordinate the immune response and resistance to tumors under certain conditions, mainly by generating tumor-specific antibodies. However, specific B cell subsets and antibody specificity can also inhibit anti-tumor immunity and promote tumor growth (Sharonov et al., 2020). Infiltrating B lymphocytes are important components of tertiary lymphoid structures (TLS) in tumor tissues. TLSs are ectopic lymphoid organs formed in non-lymphoid tissues during chronic inflammation and tumor growth that is composed of T, B, follicular dendritic, DC-LAMP^+^ dendritic, and other cells. In multiple tumor types, B cell infiltration and TLS formation are positively correlated with patient response to immunotherapy, highlighting the critical role of B cells and TLSs in anti-tumor immunity and providing a basis for new theories and strategies of tumor immunotherapy (Sautès-Fridman et al., 2019; Helmink et al., 2020). Interestingly, in the current study, the level of infiltrating memory B cells was significantly negatively correlated with NRG risk score, indicating that necroptosis also participates in B cell anti-tumor processes. This result is consistent with our finding that necroptosis is correlated with B cell receptor signaling. To our knowledge, this is the first report to document the involvement of necroptosis in B cell anti-tumor processes.

Tumor-associated macrophages (TAMs), the major component of myeloid cells in tumors, constitute two major phenotypes: M1 (inhibiting cancer progression) and M2 (promoting cancer progression) (Pan et al., 2020). TAMs exert both pro-tumor and anti-tumor effects and may therefore serve as attractive potential targets for tumor therapy. Zhao and co-workers reported that TAMs isolated from gastric cancer tissues predominantly display an M2 phenotype (Li et al., 2019) and gastric cancer-derived mesenchymal stromal cells promote metastasis and epithelial-mesenchymal transition (EMT) by triggering M2 TAM polarization through the IL-6/IL-8-JAK2-STAT3 signaling pathway. Moreover, blockage of M2 TAMs could reactivate CD8^+^ T cells against immunosuppressive tumors (Viitala et al., 2019) and infiltrating levels of M2 TAMs in gastric cancer were associated with the 5-year survival rate (Junttila et al., 2020). Here, we observed increasing infiltration of M2 TAMs in the high necroptosis risk_score group, suggesting that necroptosis may participate in the antitumor immune response via regulation of macrophages. However, further research is warranted to establish precise molecular mechanisms.

Immunotherapy has become an indispensable element of gastric cancer treatment. ICIs are an important aspect of immunotherapy, including anti-PD1 and anti-CTLA4 antibodies, which have continuously improved the survival of gastric cancer patients and progressed from back-line to front-line status in clinical practice. Several researchers have focused on the selection of effective immunotherapy biomarkers to date. MSI-H/dMMR is generally recognized as a good predictive biomarker in gastrointestinal tumors. In previous studies, MSI-H accounted for 19.09% of GC cases in a TCGA cohort and 5.75% cases in a Chinese cohort, which were higher relative to the proportion of MSI-H in other solid tumor types (Bonneville et al., 2017; Zang et al., 2019). Previous KEYNOTE-061 and KEYNOTE-062 clinical trials reported higher OS, progression-free survival (PFS), and objective response rate (ORR) with anti-PD1 therapy than chemotherapy for MSI-H gastric cancer patients (Shitara et al., 2018; Shitara et al., 2020). In the present investigation, MSI-H patients accounted for a higher percentage of the low-risk than high-risk group and the median risk_score of MSI-H was markedly lower than that of MSI-L/MSS groups. The majority of immune checkpoint genes were differentially expressed between the high- and low-risk necroptosis groups, including PD-1, PD-L1, CTLA4, and LAG3, confirming the value of exploring necroptosis in new immunotherapy approaches targeting other checkpoints. The tumor cell-killing function of immune cells is known to depend on efficient antigen presentation by human leukocyte antigen (HLA) molecules. Accumulating evidence suggests that HLA serves as a useful predictor of the efficacy of immunotherapy and HLA typing before treatment is an informative step for therapy (Chowell et al., 2018). In our experiments, HLA-DRB5 displayed distinct expression patterns among different necroptosis risk groups, supporting its potential utility in predicting response to ICB and designing neoantigen-based therapeutic vaccines in the future. We conclude that high necroptosis risk_score is correlated with low MSI-H percentage, low expression of immune checkpoints, low TMB score, and effector immune cells in GC. Precision therapy relies on accurate typing and a comprehensive understanding of the underlying mechanisms. Given the finding that necroptosis is a biological process with a relatively central role in the development of gastric cancer as well as regulation of TIME, exploration of the efficacy of targeted necroptosis therapy alone or in combination with immunotherapy should be a focus of future studies.

Wang and Liu, (2021) have identified a prognostic signature based on necroptosis hub genes in GC and uncovered a lncRNA-miRNA regulatory axis related to necroptosis, but they did not assess the TIME and immunotherapy response in GC patients. In this study, we conducted a comprehensive investigation of the immune microenvironment in GC for the first time. However, our study had several limitations that should be taken into consideration. Data was collected from public databases and required verification in clinical samples. This was a retrospective study design, which may have led to selection bias in variables and samples, and patient sample volumes were limited. Prognostic necroptosis genes combined with clinical validation in the patients of GC prospective cohort is needed to prove its efficacy. Finally, immunotherapy response was predicted according to public database website and a urothelial carcinoma cohort (Imvigor210) or melanoma cohort (GSE78220), and GC cohorts treated by immunotherapy in future research is needed.

In conclusion, we have identified an efficacious prognostic model based on necroptosis-related genes in GC and comprehensively analyzed the relationship between necroptosis and tumor immunity-associated factors. Our results provide a promising way for exploring novel and innovative targeted and immunotherapy approaches for GC patients. In the future, we aim to examine the predictive value of necroptosis risk_score in immunotherapy for gastric cancer in clinical practice and further evaluate the molecular pathways by which necroptosis influences the immune microenvironment.

## Data Availability

The datasets presented in this study can be found in online repositories. The names of the repository/repositories and accession number(s) can be found in the article/Supplementary Material. Additional data and the code used to analyze the data can be requested by contacting the corresponding author FW, zzuwangfeng@zzu.edu.cn upon reasonable request.
