# Supplementary material for: A novel prognostic and therapeutic target biomarker based on necroptosis-related gene signature and immune microenvironment infiltration in gastric cancer
Source: Front Genet. 2022 Aug 25;13:953997. doi: 10.3389/fgene.2022.953997 (PMC9452725; doi:10.3389/fgene.2022.953997)
Supplement: Supplementary file 1 [file DataSheet1.zip › ▓╣│Σ▓─┴╧/Supplementary Figures.docx]

**Supplementary Figures**


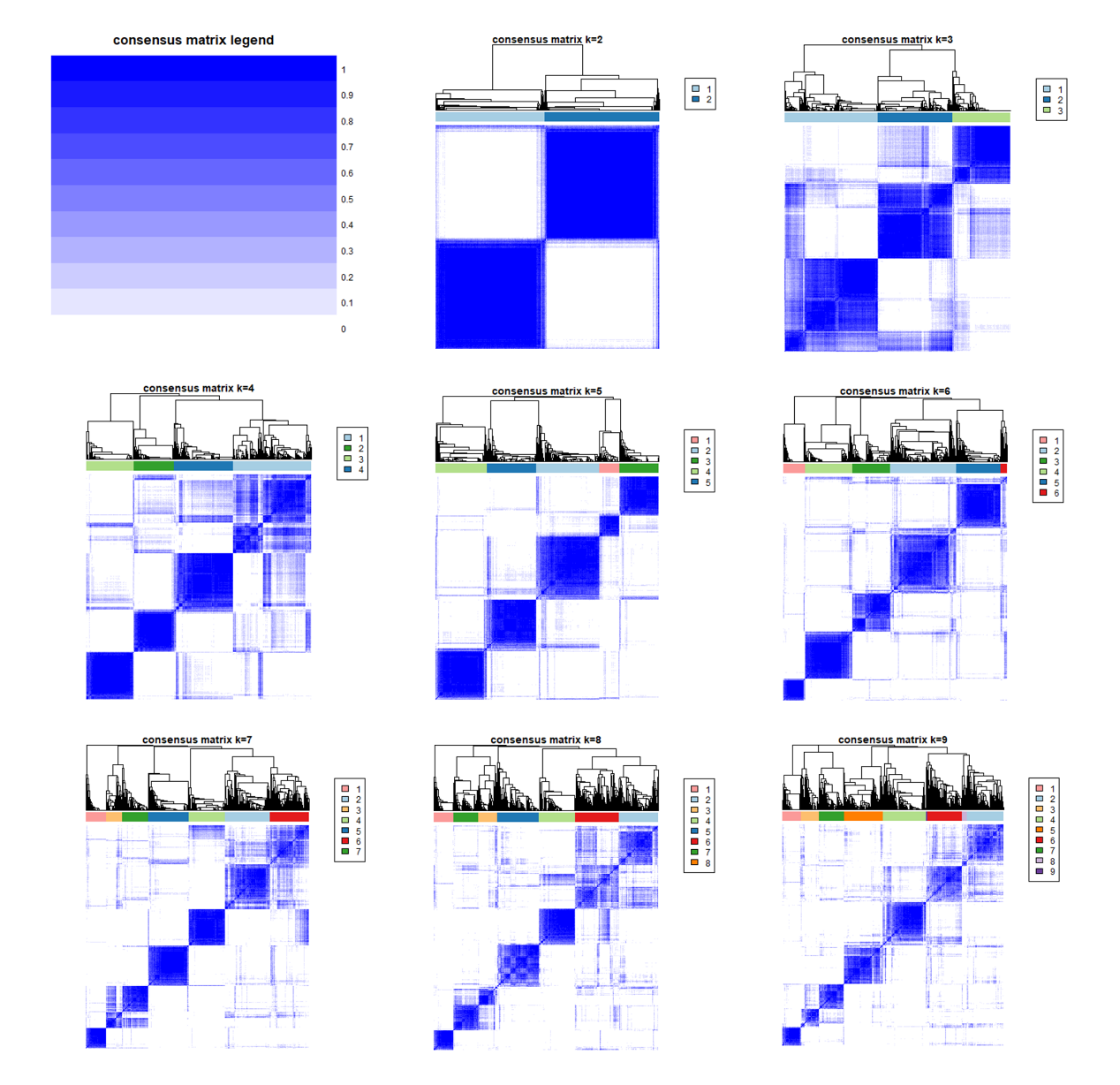


**Figure S1** Unsupervised clustering of NRGs and Consensus matrix heatmaps for k = 1-9.


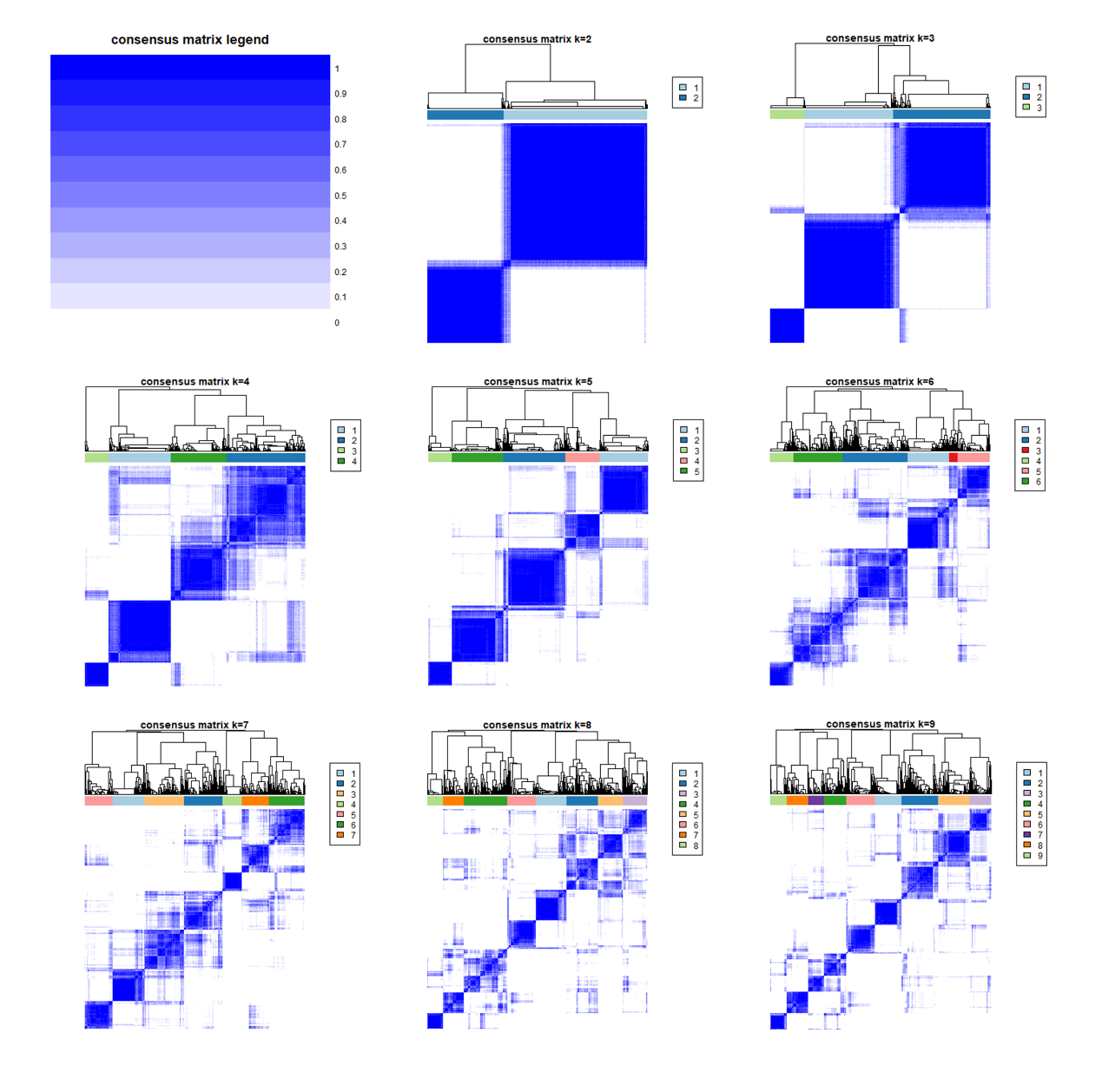


**Figure S2** Identification of necroptosis gene cluster in GC samples and consensus matrix heatmaps for k = 1-9.


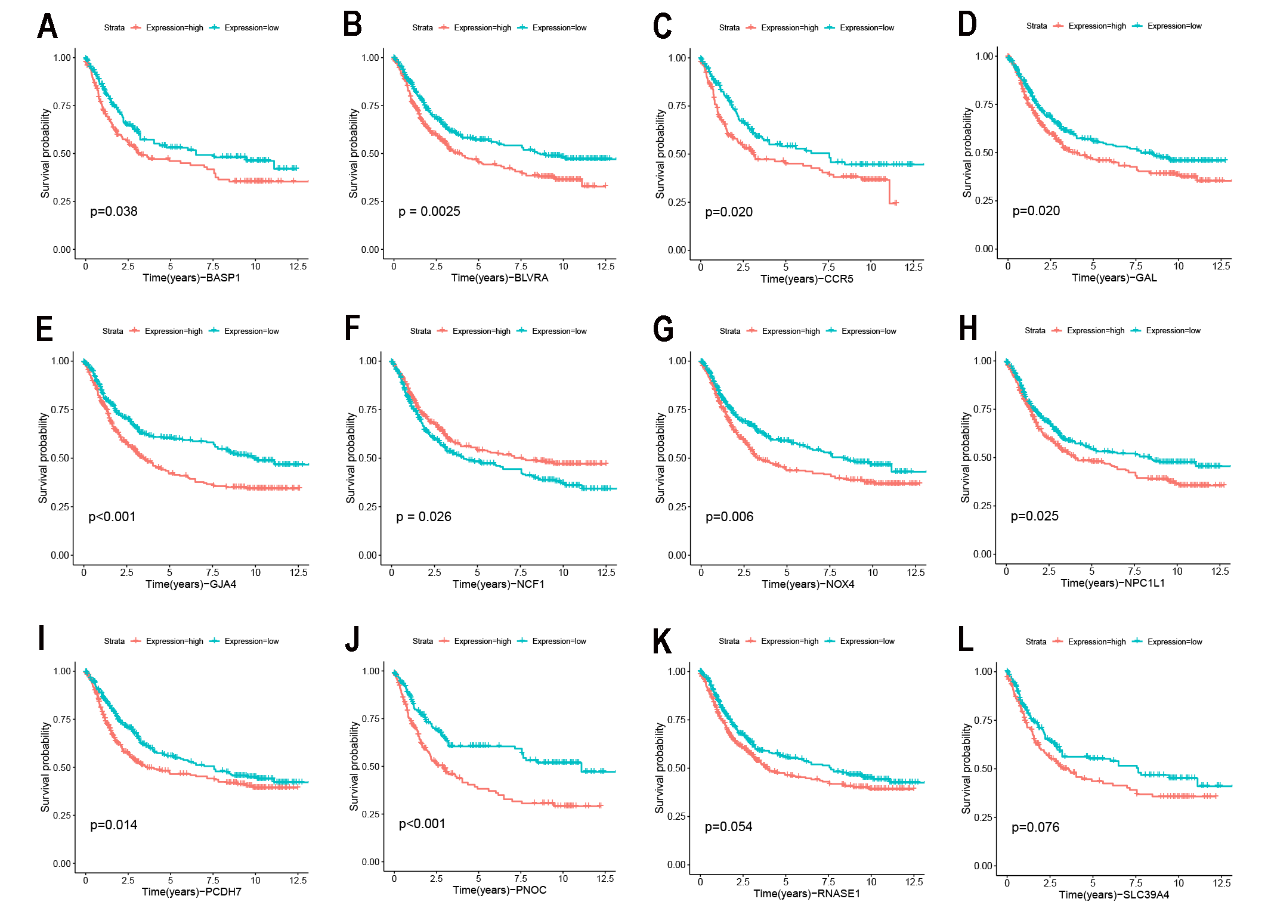


**Figure S3** Correlation of the 12 model genes expression with OS in the whole patients set


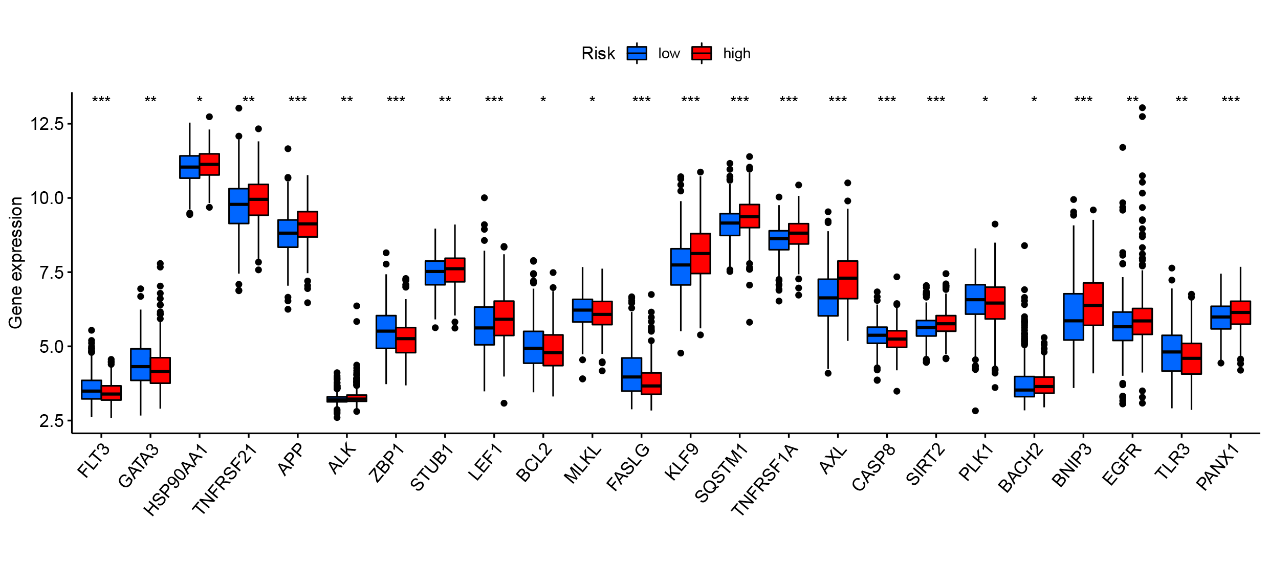


**Figure S4** NRGs expression between the high and low-risk groups.


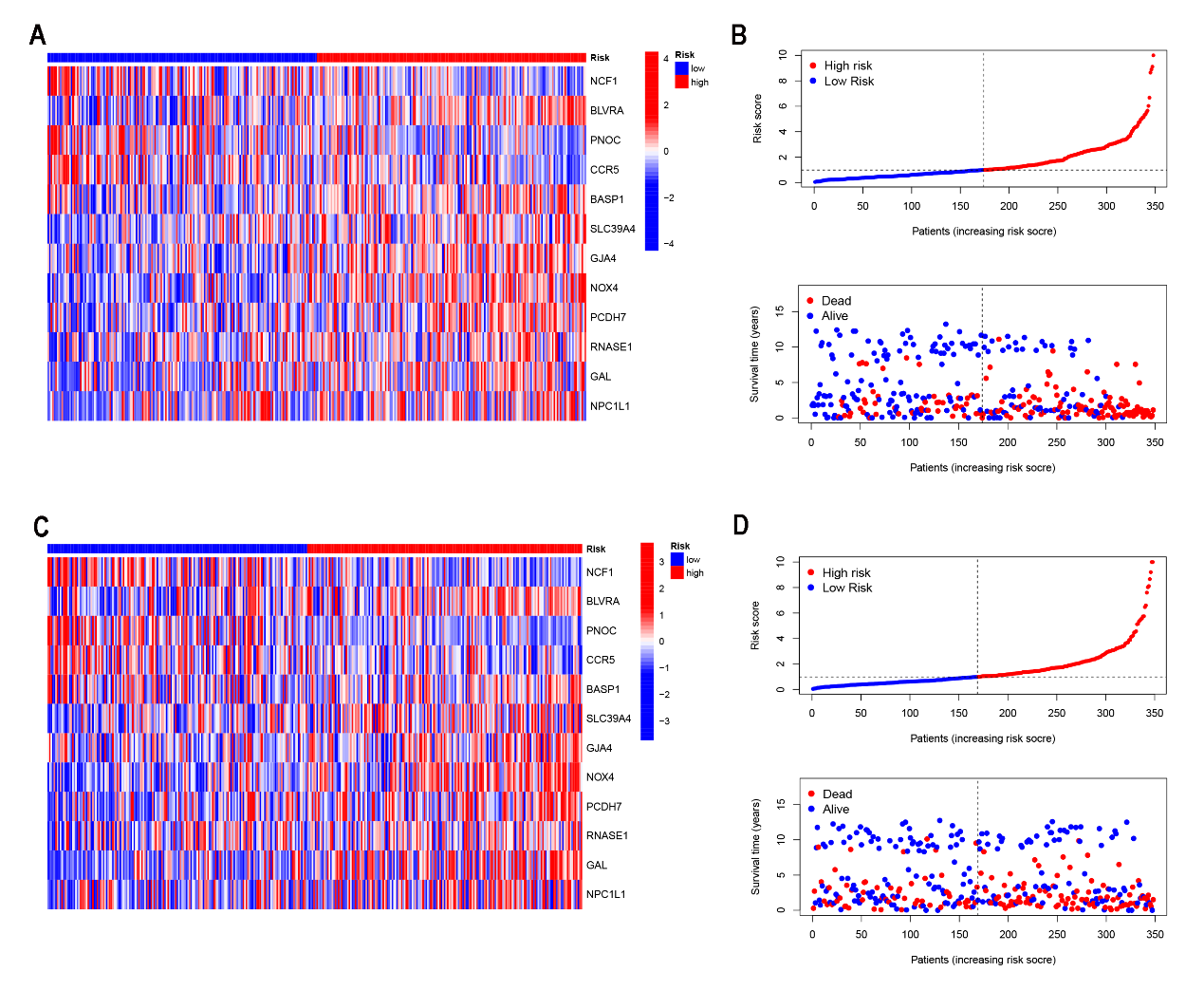


**Figure S5** Genes expression in the necroptosis risk score between the high and low-risk groups. Necroptosis risk score distribution survival status of STAD patients. (A, B in the training set and C, D in the validating set)


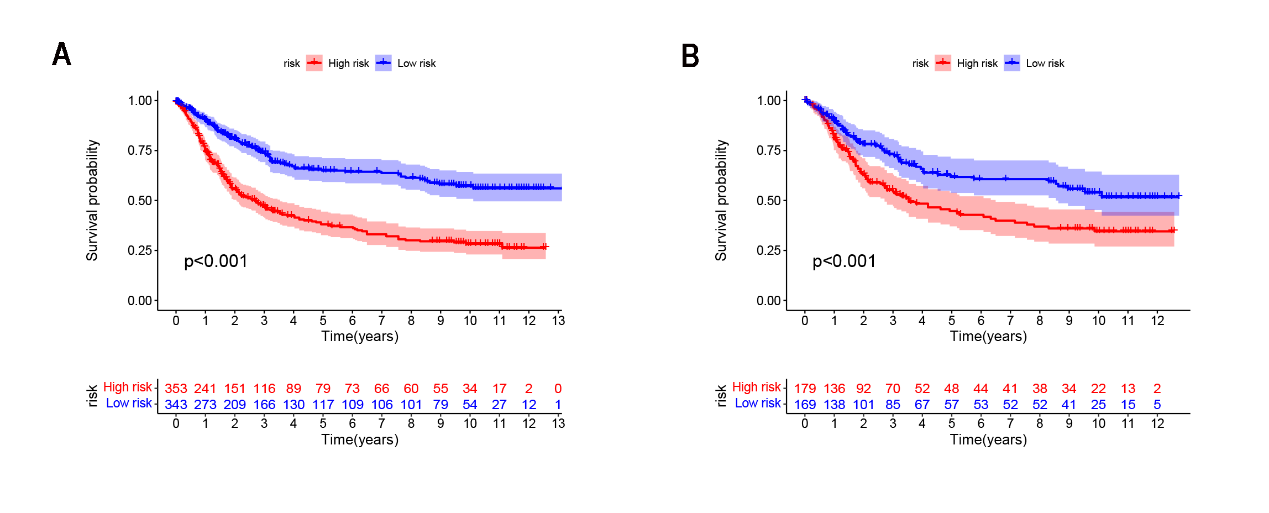


**Figure S6** Overall survival curves of risk group in the whole patient set (A) and validating set (B).


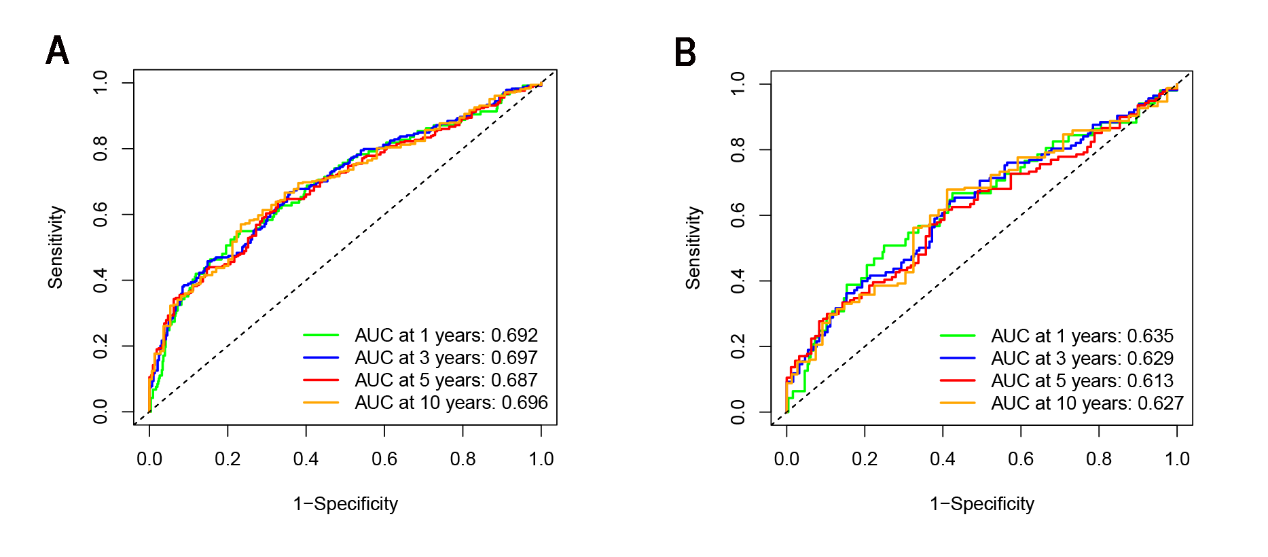


**Figure S7** ROC curves of risk group associated OS in the whole patient set (A) and validating set (B).


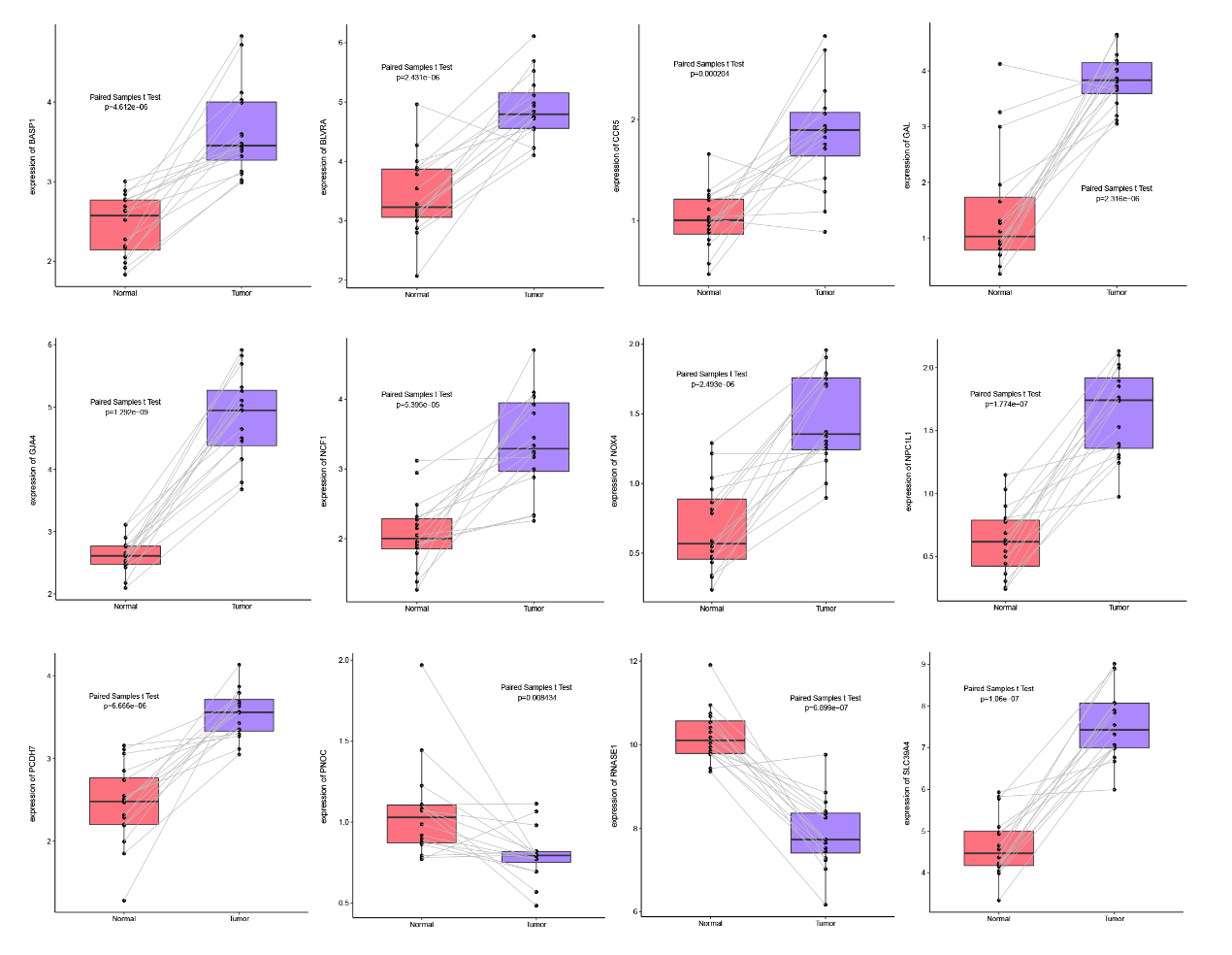


**Figure S8** Expression levels of 12 necroptosis-related genes of prognostic signature in STAD tissues and corresponding normal tissues by RT-PCR.


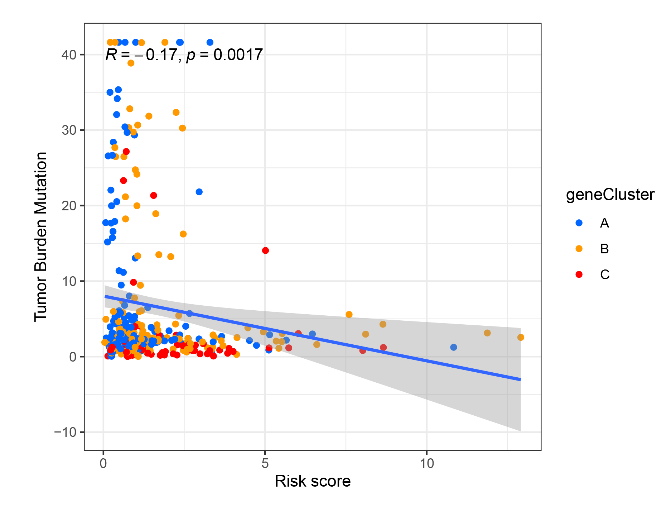


**Figure S9** Correlation of TMB score with necroptosis gene clusters.
